# Human Pluripotent Stem Cell‐Derived Skeletal Muscle Organoid Model of Aging‐Induced Sarcopenia

**DOI:** 10.1002/jcsm.70045

**Published:** 2025-08-14

**Authors:** Seongjun Park, Min‐Kyoung Shin, Dong Seok Jeong, Xin Yi Yeo, Yeo Jin Kim, Junaid Muhammad, Minju Kim, Seongeun Gu, Jeoung Eun Lee, Yunjin Park, Su Bin Lim, Ji Young Mun, Sangyong Jung, Dong Ryul Lee, Junghyun Jo

**Affiliations:** ^1^ Department of Biomedical Science, College of Biological Science CHA University Seongnam‐si Gyeonggi‐do Republic of Korea; ^2^ Department of Pharmacology Ajou University School of Medicine Suwon Gyeonggi‐do Republic of Korea; ^3^ Center for Convergence Research of Neurological Disorders Ajou University School of Medicine Suwon Gyeonggi‐do Republic of Korea; ^4^ Department of Psychological Medicine, Yong Loo Lin School of Medicine National University of Singapore Singapore; ^5^ Department of Medical Science, College of Medicine CHA University Seongnam‐si Gyeonggi‐do Republic of Korea; ^6^ Neural Circuit Research Group Korea Brain Research Institute Daegu Republic of Korea; ^7^ Department of Biochemistry & Molecular Biology Ajou University School of Medicine Suwon Gyeonggi‐do Republic of Korea; ^8^ BK21 R&E Initiative for Advanced Precision Medicine, Department of Biomedical Sciences Ajou University School of Medicine Suwon Gyeonggi‐do Republic of Korea; ^9^ CHA R&D Institute, CHA Bundang Medical Center CHA University Seongnam‐si Gyeonggi‐do Republic of Korea; ^10^ Department of Biochemistry CHA University School of Medicine Seongnam‐si Gyeonggi‐do Republic of Korea

**Keywords:** aging, human pluripotent stem cell, human skeletal muscle organoid, sarcopenia, satellite cell, testosterone

## Abstract

**Background:**

Sarcopenia is defined by the age‐related loss of muscle mass and function, with an impaired regenerative capacity of satellite cells (SCs). Despite their recognized importance in muscle regeneration, human model‐based studies on SCs in sarcopenia are still lacking, limiting our understanding of their role in age‐related muscle loss. Here, we aimed to develop a sarcopenia model using human pluripotent stem cells (hPSCs)‐derived skeletal muscle organoids (hSkMOs) and prevent the sarcopenia progression by testosterone treatment.

**Methods:**

The 3D hSkMOs were generated from hPSC and exhibited structurally and functionally mature muscle fibres and spinal‐derived neurons including motor neurons and interneurons. The proportion of muscle and the diameter of muscle fibres were assessed. To investigate the acute pro‐inflammatory response and intrinsic regenerative capacity of hSkMOs, we induced sarcopenia‐like conditions by TNF‐α treatment for 2 days and analysed. To model aging‐induced sarcopenia and investigate the preventive effect of testosterone, chronic TNF‐α treatment was applied, followed by testosterone administration. Histological, biochemical, molecular and electrophysiological analyses were conducted in various experiments.

**Result:**

We employed a stepwise differentiation protocol from 2D paraxial mesodermal induction to 3D myogenic specification, concluding with a maturation culture system. We observed that the majority of cells were T/BRA‐ and TBX6‐positive (^+^) paraxial mesodermal progenitors (T/BRA^+^, 82.04%; TBX6^+^, 78.18%), whereas the neuromesodermal progenitors demonstrated a relatively low proportion (T/BRA^+^/SOX2^+^, 15.91%; TBX6^+^/SOX2^+^, 11.45%). Single‐nucleus RNA‐sequencing and extensive immunohistochemistry confirmed the presence of the myogenic lineage cell types (myogenic progenitors/SCs, myocytes, muscle fibres) and the neural lineage cell types (spinal‐derived interneurons, motor neurons, glial cells, Schwann cells). Additionally, the growth of MyHC^+^ muscle fibres reached twice the thickness on Day 100 compared to that on Day 50 (*p* < 0.0001). We subjected them to TNF‐α treatment and analysed. Western blot analysis confirmed that TNF‐α/NF‐*κ*B pathway associated factors such as NF‐*κ*B p65, I*κ*B‐α and AKT were highly phosphorylated (*p* < 0.05, *p* < 0.001). The administration of testosterone increased the proportion of activated SCs (PAX7^+^/MYOD^+^, 7.97%; PAX7^+^/Ki67^+^, 7.03%) compared to the TNF‐α group (PAX7^+^/MYOD^+^, 2.29%; PAX7^+^/Ki67^+^, 2.07%, *p* < 0.001). The administration of testosterone increased the Cross‐Sectional‐Area (987.1 μm^2^) compared to the TNF‐α group (644.7 μm^2^, *p* < 0.01).

**Conclusions:**

We successfully developed a hSkMOs to demonstrate the structural maturity of the skeletal muscle and its functional interaction with spinal‐derived interneurons and motor neurons. Furthermore, we demonstrated that our hSkMOs are useful for modelling aging‐induced sarcopenia and providing a valuable platform for testing therapeutic interventions.

## 1 Introduction

Sarcopenia is defined as the age‐dependent loss of skeletal muscle mass and strength, which has a progressive negative impact on quality of life [1, 2]. The primary cause of sarcopenia is aging, with contributing factors such as decreased physical activity, nutritional deficits and chronic inflammation [2]. Mechanistically, sarcopenia is driven by the impaired regenerative capacity of muscle stem cells, also known as satellite cells (SCs), leading to a reduced ability to repair muscle fibres following injury [3]. Although the clinical importance of sarcopenia is being increasingly recognized, research in this area continues to face several challenges. A primary limitation is the reliance on animal models, which, while providing valuable insights, often do not fully replicate the specific molecular and cellular mechanisms driving sarcopenia in humans [4]. Additionally, studying age‐related muscle degeneration in animals is time‐consuming and resource‐intensive because of its slow progression [5, 6]. This inefficiency has hampered efforts to investigate disease development and test therapeutic interventions. Moreover, models that capture human‐specific biological signatures, which are crucial for accurately understanding the condition and developing effective treatments, are lacking.

To overcome these limitations, three‐dimensional (3D) organoid models have emerged as transformative platforms for sarcopenia research. Organoids are human stem cell‐derived, self‐organized tissues that recapitulate the architecture and functionality of native tissues. In the context of skeletal muscle research, human skeletal muscle organoids (hSkMOs) are a powerful tool for modelling muscle development, function and degeneration in vitro. Several studies have reported the generation of 3D skeletal muscles from human pluripotent stem cells (hPSCs) to study skeletal muscle development and regenerative capacity [7, 8, 9, 10]. These organoids offer distinct advantages, including the ability to replicate human‐specific features, and provide a controlled environment for disease progression. Although the embryonic development of skeletal muscle tissue can be recapitulated using these methods, reports on muscle fibres, including mature sarcomeres, are few. Therefore, the study of sarcopenic muscle using hPSC‐derived 3D hSkMO remains challenging. In our previous studies, we successfully generated hSkMOs that replicated critical aspects of skeletal muscle development and muscle regenerative capacity of SCs [10].

Here, we aimed to develop a sarcopenia model in advanced hSkMOs by inducing muscle atrophy and wasting through repeated tumour necrosis factor‐alpha (TNF‐α) exposure. TNF‐α, a pro‐inflammatory cytokine, promotes muscle degradation under chronic inflammatory conditions, primarily through activation of the AKT–mTOR and nuclear factor kappa B (NF‐*κ*B) signalling pathways [11]. This approach allowed the establishment of a robust in vitro model of sarcopenia that reflects chronic muscle degeneration. Additionally, we investigated the effects of testosterone as a potential therapeutic intervention to counteract muscle atrophy through its anabolic effects. Testosterone helped mitigate muscle wasting within the TNF‐α‐induced sarcopenia model, offering insights into hormone‐based strategies for the prevention of age‐related muscle loss.

## 2 Methods

### 2.1 Generation of hSkMOs

The hPSCs were dissociated into single cells using TrypLE. Single cells were counted and were plated on a 12‐well plate coated with matrigel (Corning) at a density of 62 500–75 000/cm^2^. At first day, the cells were plated in mTeSR medium (Stem Cell Technologies) supplemented with 1 μM Rock inhibitor (Tocris Bioscience). The next day, Rock inhibitor was removed, and the cells were differentiated in paraxial mesoderm induction medium (PIM) supplemented with 3 μM CHIR99021 (Stem Cell Technologies) and 0.5 μM LDN193189 (Stemgent) until Day 3. PIM consists of 1% insulin‐transferrin‐selenium (ITS; Life Technologies), 1% nonessential amino acids (NEAA; Thermo Fisher Scientific) and 0.25% penicillin/streptomycin (P/S; Gibco) in DMEM/F12 (Gibco). The medium was changed every day. Differentiated paraxial mesodermal progenitors (PMPs) were dissociated using TrypLE to generate a single cell suspension. On Day 3, PMPs (5000–10 000/well) were plated on an ultra‐low binding V‐bottom 96‐well plate (Corning) in PIM medium with 1 μM Rock inhibitor, 3 μM CHIR99021 and 0.5 μM LDN193189 and 20 ng/mL bFGF (Peprotech). The initial volume in each well was 100 μL. The next day, Rock inhibitor was removed, and the cells were maintained in PIM medium supplemented with 3 μM CHIR99021, 0.5 μM LDN193189 and 20 ng/mL bFGF until Day 6. The medium was changed every day. On Day 6, the hSkMOs were cultured in the myogenic differentiation medium (MDM) supplemented with 10 ng/mL HGF (Peprotech), 2 ng/mL IGF1 (Sigma‐Aldrich) and 20 ng/mL bFGF until Day 9. MDM consists of 15% KSR (Thermo Fisher Scientific), 1% NEAA, 1% P/S and 0.1 mM β‐mercaptoethanol in DMEM/F12. On Day 9, the hSkMOs were transferred to a six‐well plate on an orbital shaker and cultured in MDM supplemented with 10 ng/mL HGF and 2 ng/mL IGF1. On Day 30, the hSkMOs were cultured in MDM supplemented with 5 μM SB431542 (Sigma) until Day 50. From Day 50, hSkMOs were cultured in myogenic maturation medium (MMM), which consists of 1% N2 (Gibco), 2% B27 without vitamin A, 1% GlutaMAX, 1% NEAA, 1% P/S and 0.1% β‐mercaptoethanol in DMEM/F12.

### 2.2 Quantification and Statistical Analyses

Data are reported as the mean ± standard error (*n* numbers are described in the figure legends) of the mean, using a significance level of *p* < 0.05. Data were analysed by *t*‐test and one‐way ANOVA using GraphPad Prism software V10.

## 3 Results

### 3.1 Efficient Generation of hPSC‐Derived 3D hSkMOs Through Stepwise Differentiation

**FIGURE 1:**
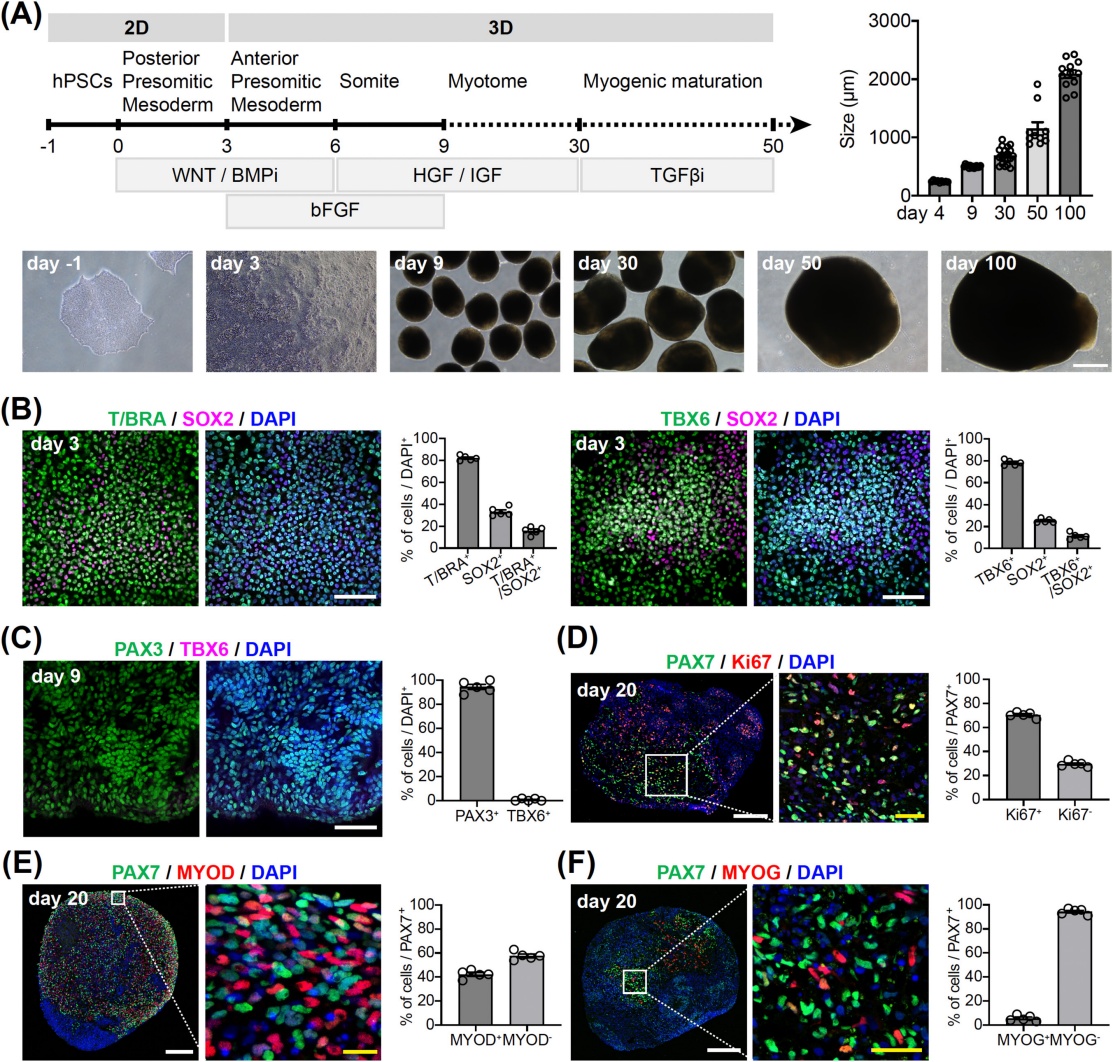
Efficient generation of hPSC‐derived 3D hSkMOs. (A) A schematic diagram showing the overall strategy to generate hSkMOs from hPSCs. The representative images of hSkMOs morphology are shown at each time point. Scale bar = 500 μm. The growth diameter demonstrates the average size (mean ± SEM; *n* = 10). (B) Immunocytochemistry analyses of Day 3 PMPs stained for T/BRA, TBX6, SOX2 and DAPI (mean ± SEM; *n* = 5). Scale bars = 200 μm. (C) A whole mount staining image of a Day 9 hSkMOs stained for PAX3, TBX6 and DAPI (mean ± SEM; *n* = 5). Scale bar = 100 μm. (D) Cryosection of Day 20 hSkMOs stained for PAX7, Ki67 and DAPI (mean ± SEM; *n* = 5). White scale bar = 200. Yellow scale bar = 20 μm. (E) Cryosection of Day 20 hSkMOs stained for PAX7, MYOD and DAPI (mean ± SEM; *n* = 5). White scale bar = 200. Yellow scale bar = 10 μm. (F) Cryosection of Day 20 hSkMOs stained for PAX7, MYOG and DAPI (mean ± SEM; *n* = 5). White scale bar = 200. Yellow scale bar = 20 μm.

Previously, we reported a method for generating hSkMOs from hPSCs to demonstrate the time‐course features of skeletal muscle development and the regenerative capacity of sustainable SCs upon chemical damage [10]. Although our direct induction approach for paraxial mesodermal differentiation and subsequent myogenic specification successfully achieved the features of skeletal muscle development, the culture system required further refinement to efficiently produce hSkMOs. Therefore, we optimized and modified our previous method to differentiate more homogenous PMPs. We reasoned that the acquisition of more potent and homogenous PMPs is important for efficiently inducing presomitic mesoderm (PSM) specification. To this end, we employed a stepwise differentiation protocol starting from two‐dimensional (2D) paraxial mesodermal induction, transitioning to 3D myogenic specification, and concluding with a maturation culture system. The hSkMOs gradually grew to approximately 2 mm in diameter by Day 100 (Figure 1A).

First, the hPSCs were dissociated into single cells and seeded at 2 × 10^5^ cells/well in 12‐well plates. The following day, the cells were exposed to a WNT activator (CHIR99021) and BMP inhibitor (LDN193189) to promote paraxial mesodermal differentiation for 3 days. We observed a rapid upregulation of key transcriptional regulators of posterior presomitic mesoderm (pPSM) specification such as *T/Brachyury*, T‐Box transcription factor 6 (*TBX6*) and *MSGN1* on Day 3 (Figure S1A). To further characterize the differentiated PMPs, we performed immunocytochemical analysis on Day 3 using antibodies against paraxial mesodermal and neuromesodermal progenitor markers. We observed that the majority of cells were T/Brachyury‐ and TBX6‐positive (^+^) paraxial mesodermal cells (T/Brachyury^+^, 82.04%; TBX6^+^, 78.18%) (Figure 1B), whereas the double‐positive SOX2 neuromesodermal cells demonstrated a relatively low proportion (T/Brachyury^+^ and SOX2^+^, 15.91%; TBX6^+^ and SOX2^+^, 11.45%) (Figure 1B). The somite develops through anterior presomitic mesoderm (aPSM) specification from the paraxial mesodermal cells [12, 13, 14]. Therefore, the PMPs were dissociated and plated in V‐bottom ultra‐low‐adhesion 96‐well plates to transit to the 3D culture system (Figure 1A) supplemented with fibroblast growth factor 2 (FGF2) with continuous CHIR99021 and LDN193189 treatment. At Day 6, the organoids were subjected to hepatocyte growth factor (HGF) and insulin‐like growth factor (IGF) to promote somite specification (Figure 1A). Consequently, we observed that the organoids displayed upregulation of somitogenesis markers such as *PAX3* and *MEOX2* (Figure S1A). Immunohistochemical analysis further revealed that most cells were PAX3^+^ (Figure 1C); however, the expression of *T/Brachyury*, *TBX6*, and *MSGN1* was drastically decreased (Figure 1C, S1A). These data indicate that our hSkMOs successfully recapitulated the essential early commitment of the developmental stages in paraxial mesodermal differentiation, transitioning from pPSM to aPSM and somite formation.

Next, the organoids were transferred to six‐well tissue culture plates and cultured on an orbital shaker from Day 9 until the day of analysis (Figure 1A). As we previously demonstrated, the use of an orbital shaker significantly improved the culture condition of the organoids [10, 15, 16]. The organoids were cultured with HGF and IGF for myogenic proliferation and differentiation, and FGF2 treatment was omitted (Figure 1A). To characterize the developing hSkMOs, we performed immunohistochemical analysis using antibodies against myogenic progenitors on Day 20. We observed that 70.41% of PAX7^+^ cells were co‐labelled with Ki67 (Figure 1D). Additionally, approximately 42.30% of PAX7^+^ myogenic progenitors were double‐positive for MYOD, whereas the remaining 57.70% of cells did not express MYOD (Figure 1E). Furthermore, 5.91% of the PAX7^+^ cells were co‐labelled with MYOG, demonstrating the proliferation, activation and differentiation of PAX7^+^ myogenic progenitors in hSkMOs (Figure 1F). Additionally, although the hSkMOs are primarily committed to the paraxial mesodermal and myogenic lineage specification, TFAP2A^+^ neural crest cells and SOX2^+^ neural progenitor cells were also observed. This indicates a partial capacity for neuromuscular lineage cells giving rise to both skeletal muscle and spinal cord neuronal cells (Figure S1B,C). Taken together, these data demonstrate that our in vitro hSkMOs recapitulate the stepwise developmental features of paraxial mesoderm, presomite and somite differentiation.

### 3.2 Mature hSkMOs Exhibit Structurally Organized Muscle Fibres, quiescent SCs and Functional Neuromuscular Junctions

**FIGURE 2:**
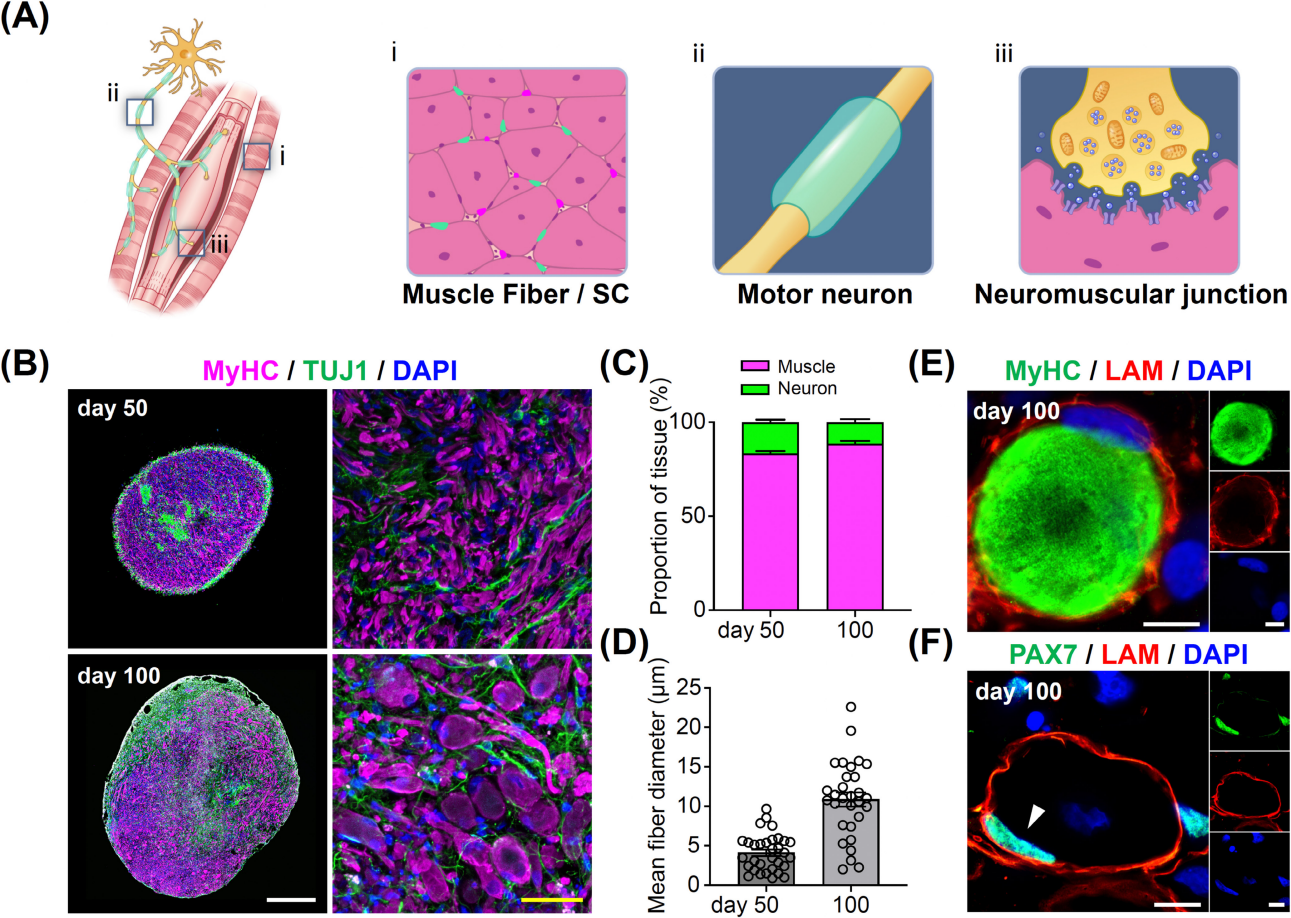
Structural characterization of mature muscle fibres in hSkMOs. (A) A schematic illustration of mature muscle fibres and functional connection with spinal‐derived motor neurons. (B) Cryosection of Days 50 and 100 hSkMOs stained for MyHC, TUJ1 and DAPI. White scale bar = 500. Yellow scale bar = 100 μm. (C) Quantification of the proportion of TUJ1^+^ neural and MyHC^+^ muscle region at day 50 and 100 (mean ± SEM; *n* = 14). (D) Quantification of the mean fibre diameter stained for MyHC (mean ± SEM; *n* = 32). (E) Cryosection of Day 100 hSkMOs stained for MyHC, LAMININ and DAPI. Scale bar = 10 μm. (F) Cryosection of Day 100 hSkMOs stained for PAX7, LAMININ and DAPI. Scale bar = 10 μm.

hSkMOs constituted the majority of dermomyotomal myogenic cells and a small proportion of neuromesodermal neural cells [10]. The spatial and structural organization of muscle fibres and functional connection to spinal‐derived motor neurons by neuromuscular junctions (NMJs) are critical features of skeletal muscle tissue (Figure 2A) [17]. During skeletal muscle development, myogenic cells are activated in response to various signals, which lead to their proliferation and differentiation into myocytes. These myocytes align and fuse to form multinucleated myotubes, which are the initial stage of muscle fibre formation (Figure 2A) [18].

To investigate the anatomical skeletal muscle structure of hSkMOs, we assessed the expression of key markers to evaluate both the muscle and neuronal lineages on Days 50 and 100. Immunohistochemical analysis revealed robust expression of myosin heavy chain (MyHC; mature myotube marker) and TUJ1^+^ (a neuronal marker), which were observed alongside muscle fibres in both Days 50 and 100 hSkMOs (Figure 2B,C). Notably, the growth of MyHC^+^ muscle fibres continued and reached twice the thickness on Day 100 compared to that on Day 50, indicating that long‐term culture promotes greater maturity of muscle fibres (Figure 2D). To further assess the reproducibility of our hSkMO differentiation protocol across genetically distinct hPSC lines, we generated hSkMOs from both H9 human embryonic stem cell (hESC) line and CHA‐SCNT‐18 hESCs (human somatic cell nuclear transfer embryonic stem cell) line using the same protocol. The resulting organoids exhibited comparable morphologies and growth rates across Days 10, 30, 50 and 100, revealing no significant differences in organoid size between lines (Figure S2A). At Day 30, PAX7^+^ SCs and MYOD^+^ activated SCs were detected in both lines, and the proportion of PAX7^+^/MYOD^+^ activated SCs was 45.84% in H9 and 46.99% in CHA‐SCNT‐18, which were similar proportions of the activated SCs compared to H1 hESC‐derived hSkMOs, indicating robust and reproducible establishment of functional SC populations across hPSC lines (Figure S2B). At Day 100, TUJ1^+^ neurons and MyHC^+^ muscle fibres were readily observed in the hSkMOs derived from both hPSC lines, and the neuron‐to‐muscle ratio also confirmed no significant difference between cell lines, further supporting the consistent differentiation into neuromuscular components (Figure S2C). Together, these data underscore the reproducibility of our method across hPSC lines in generating hSkMOs with conserved cellular composition and lineage architecture.

SCs are stem cells that play a key role in the growth, repair, and regeneration of skeletal muscle. They are located between the basal lamina and sarcolemma of the muscle fibres [18, 19]. To define the structural maturity of the muscle fibre, we performed immunohistochemical analysis using antibodies against human laminin. The MyHC^+^ muscle fibres were surrounded by a continuous sheath of basal lamina labelled with human laminin, indicating the establishment of a mature basal lamina structure (Figure 2E). Furthermore, PAX7^+^ SCs were located beneath the laminin‐positive basal lamina, closely recapitulating the niche of quiescent SCs observed in native skeletal muscle (Figure 2F). This spatial arrangement confirms that the PAX7^+^ SCs reside within the correct anatomical niche between the basal lamina and muscle fibres, reflecting in vivo‐like architecture. Within the skeletal muscle, various microenvironments contribute to muscle homeostasis and regeneration [20]. Fibro‐adipogenic progenitors (FAPs) play a crucial role in muscle repair by interacting with SCs and promoting muscle regeneration, while also being involved in fibrosis under pathological conditions [21]. In our hSkMOs, we confirmed the presence of PDGFRα^+^ cells, which are characteristic of FAPs within the muscle microenvironment (Figure S2D).

**FIGURE 3:**
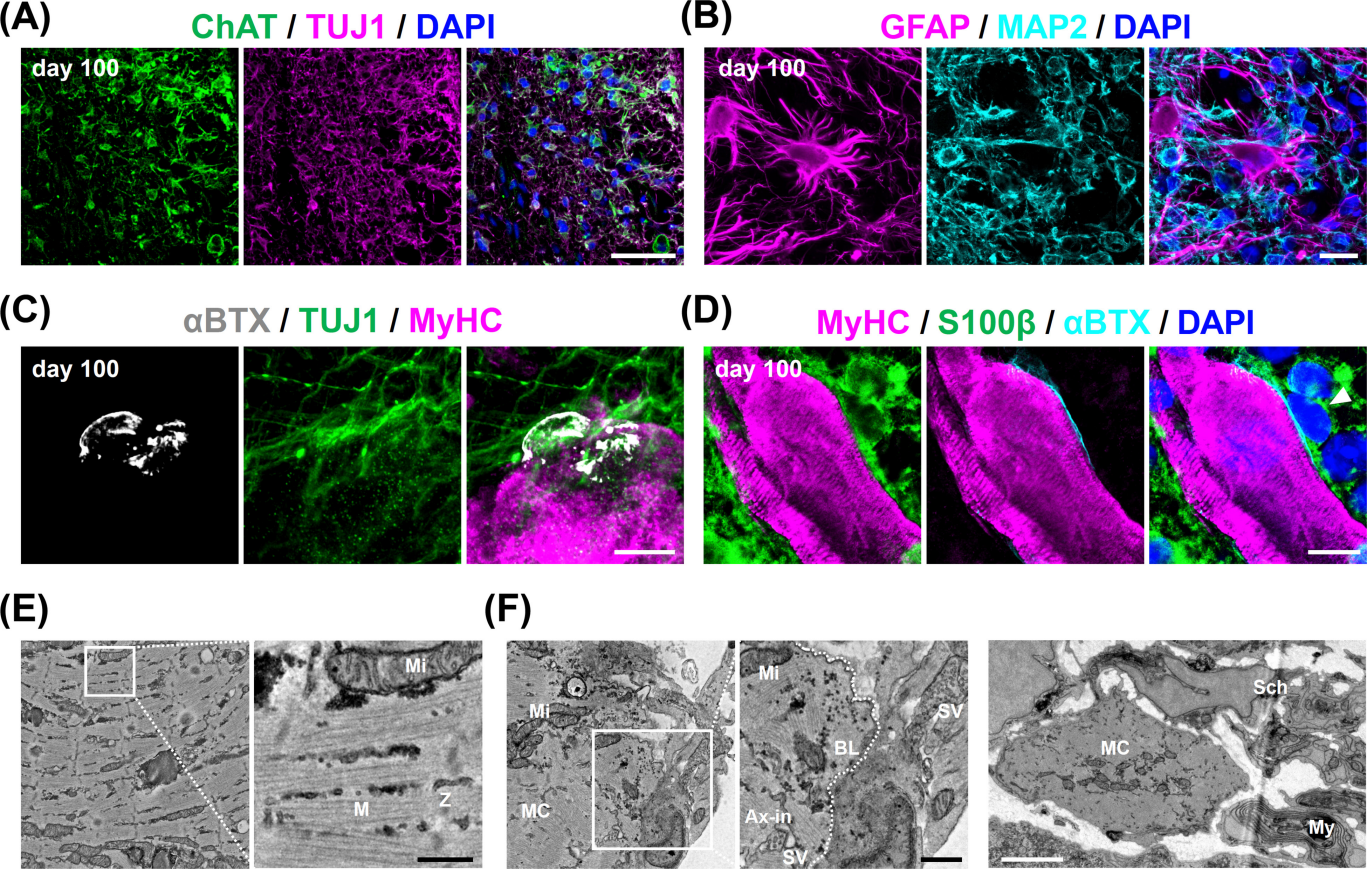
Structural characterization of spinal‐derived motor neurons and functional connection with muscle fibres in hSkMOs. (A) Cryosection of Day 100 hSkMOs stained for ChAT, TUJ1 and DAPI. Scale bar = 50 μm. (B) Cryosection of Day 100 hSkMOs stained for GFAP, MAP2 and DAPI. Scale bar = 20 μm. (C) Cryosection of Day 100 hSkMOs stained for αBTX, TUJ1 and MyHC. Scale bar = 10 μm. (D) Cryosection of Day 100 hSkMOs stained for MyHC, S100β, αBTX and DAPI. Scale bar = 10 μm. (E) and (F) Electron microscopic images to show skeletal muscle fibres of Day 100 hSkMOs. Mi: Mitochondria, M: M‐band, Z: z line., SV: Synaptic vesicles, BL: Basal lamina, Ax‐in: Axonal innervation, MC: Muscle cell, Sch: Schwann cell, My: Myelin‐like sheath. Black scale bar = 500 nm. White scale bar = 2 μm.

Next, we examined the neural lineage characteristics of hSkMOs using immunohistochemical analysis. We observed a neural proportion of approximately 10.5% in hSkMOs (Figure 2C) and further confirmed that the cells were spinal‐derived motor neurons expressing the acetylcholine (ACh)‐synthesizing enzyme choline acetyltransferase (ChAT) (Figure 3A). We also observed GFAP^+^ glial cells in hSkMOs on Day 100 (Figure 3B). Moreover, we detected α‐bungarotoxin (αBTX) immunolabelled ACh receptor clusters connected by TUJ1^+^ projections on the basal lamina of muscle fibres, indicating the formation of NMJs (Figure 3C). S100β^+^ Schwann cells were also detected around the muscle fibres (Figure 3D). The formation of NMJs on muscle fibres was verified by ultrastructural analysis using electron microscopic imaging. We observed well‐developed skeletal muscles exhibiting organized sarcomeres with distinct bands and mitochondria within compact muscle tissue (Figure 3E). Synaptic vesicles in the presynaptic axonal terminal were detected in a specific region of the basal lamina along the muscle fibres, supporting the establishment of neuromuscular connectivity (Figure 3F). Furthermore, electron microscopic images revealed the presence of Schwann cells and myelin‐like sheaths surrounding the muscle regions (Figure 3F).

### 3.3 Single‐Nucleus Transcriptomic Profiling Reveals Myogenic and Neural Cellular Diversity in hSkMOs

**FIGURE 4:**
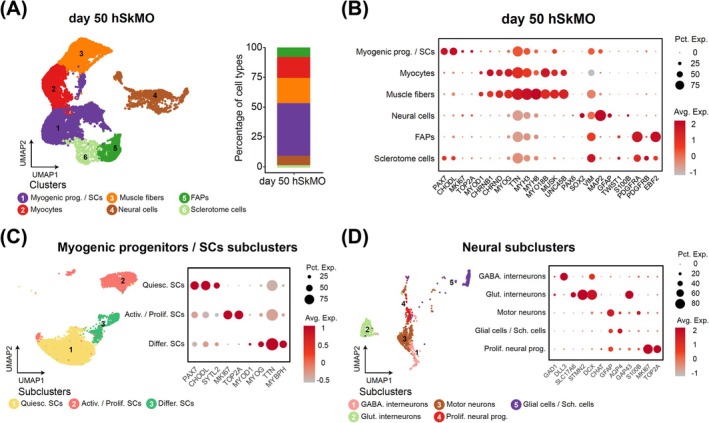
Single‐nucleus transcriptomic profiling reveals dynamic cellular heterogeneity in hSkMOs. (A) UMAP plot of snRNA‐seq data from Day 50 hSkMOs, showing distinct clusters representing major cell types including myogenic progenitors/SCs, myocytes, muscle fibres, neural cells, FAPs and sclerotome. Cell type proportions at Day 50. (B) Dot plot showing expression of representative genes in each cluster in Day 50 hSkMOs. (C) Subclustering of the myogenic progenitors/SCs identifies quiescent SCs, activated/proliferating SCs and differentiating SCs. Dot plot showing the expression of representative genes across the three subclusters. (D) Neural subclustering reveals five distinct cell populations including GABAergic interneurons, glutamatergic interneurons, motor neurons, proliferative neural progenitors and glial cells/Schwann cells. Myogenic prog.: Myogenic progenitors, Quiesc. SCs: Quiescent SCs, Activ./Prolif. SCs: Activated and proliferating SCs, Differ. SCs: Differentiating SCs, GABA. Interneurons: GABAergic interneurons, Glut. Interneuron: Glutamatergic interneurons, Prolif. neural prog.: Proliferative neural progenitor cells, Sch. cells: Schwann cells.

To systematically characterize the cellular composition and transcriptional landscape of hSkMOs, we performed single‐nucleus RNA sequencing (snRNA‐seq) on Day 50 hSkMOs (*n* = 16 278 nuclei from Day 50 hSkMOs). Uniform manifold approximation and projection (UMAP) analysis identified distinct clusters representing major cell populations, underlining the cellular diversity present within the hSkMOs. Subsequent quantification of each cell type proportion demonstrated myogenic progenitors/SCs, myocytes, muscle fibres, neural cells, FAPs and sclerotome cells comprising 43.6%, 16.8%, 21.2%, 8.0%, 7.9% and 2.2% of the total cell population, respectively (Figure 4A). To further characterize cell type identities and validate our annotations, we examined the expression profiles of canonical marker genes across the major clusters (Figure 4B, S3A). Myogenic progenitors/SCs expressed high levels of *PAX7* and *CHODL*, whereas myocytes strongly expressed *MYOG* and *MYOD1*. Terminally differentiated muscle fibres displayed robust expression of *MYH3* and *MYH8*. Neural populations were characterized by *SOX2*, *MAP2*, *GFAP* and *S100β* expression, and FAPs and sclerotome cells were identified by the expression of *PDGFRA*, *PDGFRB* and *TWIST1*, *EBF2*, respectively (Figure 4B, S3A).

SCs can be transcriptionally classified into three distinct states: quiescent, activated/proliferating and differentiating, reflecting their dynamic roles in muscle maintenance and regeneration. This classification enables detailed resolution of SC transitions during myogenesis, homeostasis and injury response. Subclustering analysis revealed these distinct SC states, resolving three transcriptionally defined subtypes: *PAX7*‐expressing quiescent SCs, *MKI67*, *TOP2A*, and *MYOD1*‐expressing activated/proliferating SCs, and *MYOG*, *TTN*, and *MYBPH*‐expressing differentiating myoblasts, representing 28.7%, 10.0% and 4.9% of the total population, respectively (Figure 4C, S3B). Considering the complexity and specialization of neural cell populations in the spinal cord connected with skeletal muscle fibres, we further subclustered neural cells to identify specific neuronal and glial subtypes. This revealed the presence of *CHAT*‐expressing motor neurons, *GAD1*‐expressing GABAergic interneurons, *SLC17A6*‐expressing glutamatergic interneurons and proliferating neural progenitors (3.3%, 0.8%, 2.0% and 0.6%, respectively) (Figure 4D, S3C). Importantly, glial cells and Schwann cells, indicated by the expression of *GFAP* and *S100β*, were also identified, reflecting spinal‐derived neuromuscular support cells critical for NMJ formation and function (Figure 4D, S3C). Collectively, these results demonstrate comprehensive cellular heterogeneity and transcriptional diversity within hSkMOs, establishing a physiologically relevant in vitro model for studying human neuro‐musculoskeletal development, ageing and disease.

### 3.4 Functional Characterization of Neuromuscular Connectivity in hSkMOs

**FIGURE 5:**
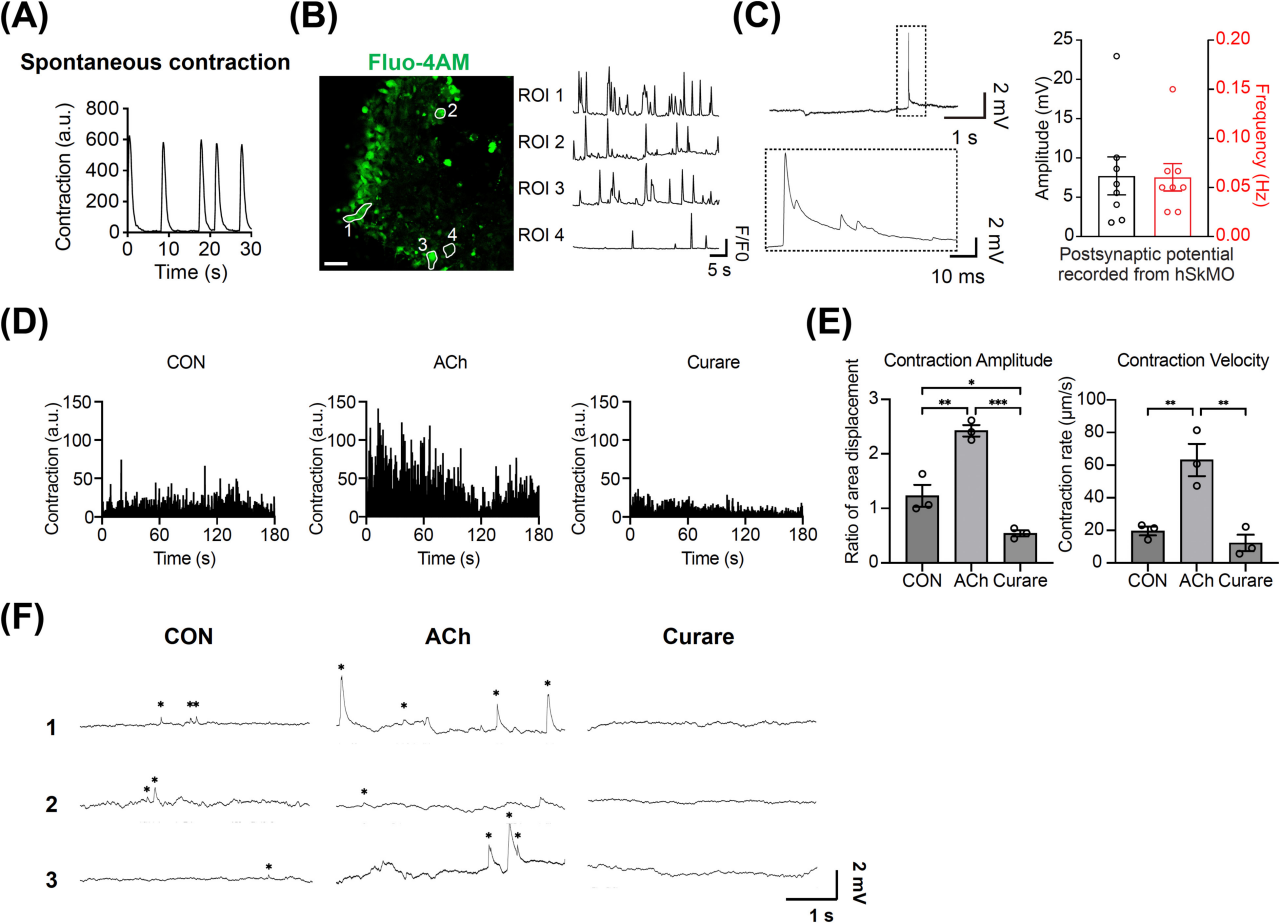
Functional characterization of hSkMOs. (A) Spontaneous contraction and (B) calcium imaging with Fluo‐4 AM for visualizing calcium influx of Day 100 hSkMOs. Scale bar = 50 μm. (C) Representative trace of the spontaneous postsynaptic potential (PSP) response obtained from the section of the organoid with muscle fibres (top). Enlarged PSP response observed (bottom left). Their amplitude and frequency (bottom right) from individual muscle fibres targeted (mean ± SEM; *n* = 8). (D) Representative trace graph depicting spontaneous contractions in mature hSkMOs, and their pharmacological modulation by acetylcholine (ACh) and curare. (E) Quantitative analysis of contraction dynamics in control, ACh‐treated, and curare‐treated hSkMO (mean ± SEM; *p < 0.05, **p < 0.01, ***p < 0.001; *n* = 3). (F) ACh and curare affect the postsynaptic potential response from the section of hSkMOs. Traces obtained from three independent cells presented and responses are marked with asterisks.

To complement our transcriptomic analyses and validate the functional integrity of hSkMOs, we next focused on their contractile behaviour and neuromuscular activity. Notably, hSkMOs exhibited spontaneous contractions during long‐term culture, suggesting the establishment of physiologically active NMJs. To further assess their functional maturity, we measured the contractile activity of the organoids, highlighting their potential as a robust model for studying neuromuscular interactions in vitro (Figure 5A). Calcium influx was observed in spinal‐derived motor neurons and muscle fibres that could be distinguished by morphology (Figure 5B). The regions of neurons or muscle fibres can be distinguished with water immersion optics for electrophysiology, and the organoids present functional muscle‐neuron connections in the form of spontaneous postsynaptic potential detected with whole‐cell patch clamp recordings from the muscle fibres (Figure 5C). To further confirm the functional competence of these NMJs, we applied acetylcholine (ACh) and curare treatments to mature hSkMOs exhibiting spontaneous contractions. Using Muscle Motion analysis, we observed that ACh treatment enhanced contraction velocity, whereas curare significantly suppressed spontaneous contractions, supporting the presence of functional acetylcholine receptor‐dependent neuromuscular transmission in our hSkMOs (Figure 5D,E). Indeed, ACh exposure increased the strength while curare inhibited the membrane potential responses observed with whole‐cell patch clamp recordings (Figure 5F). In addition, the formation of functional NMJs in the hSkMOs was confirmed by immunostaining, and electron microscopic imaging demonstrated pretzel‐like synaptic architectures of αBTX‐labelled NMJs, axonal innervation and basement membrane folding, which are characteristic of morphologically mature NMJs (Figures 3C and 4F). These results collectively demonstrate that the NMJs within hSkMOs exhibit both functional neurotransmission capacity and advanced structural maturity, reinforcing the utility of our system as a physiologically relevant model for studying human NMJs.

### 3.5 TNF‐α/NF‐*κ*B Pathway Induces Muscular Damage and Subsequent Regeneration in hSkMOs

**FIGURE 6:**
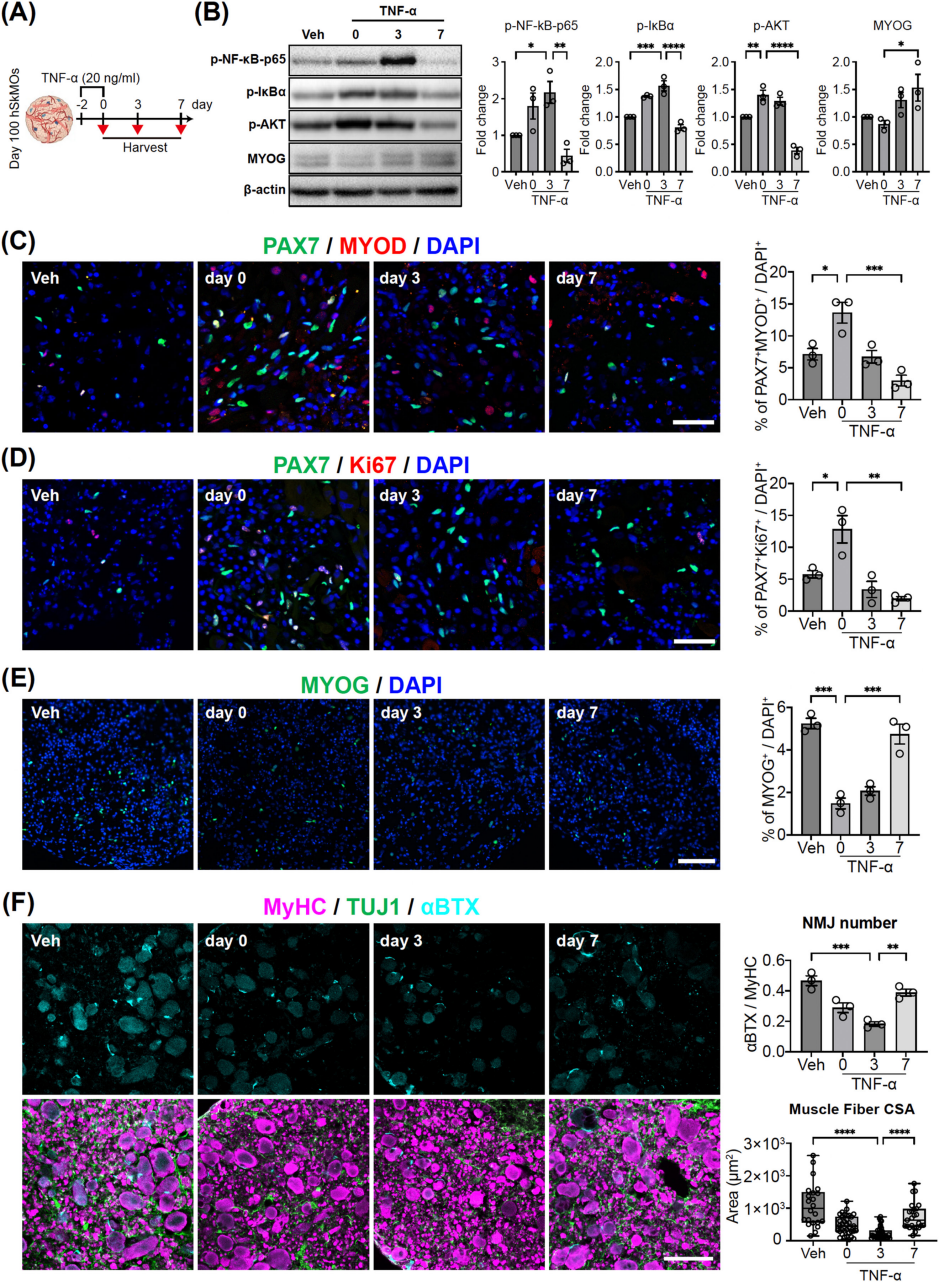
TNF‐α/NF‐*κ*B pathway‐induced muscular damage and regenerative capacity of hSkMOs. (A) A schematic diagram showing the overall strategy to acute treatment of TNF‐α on day 100 hSkMOs. (B) Western blot of p‐NF‐*κ*B‐p65, p‐I*κ*B‐α, p‐AKT, MYOG and β‐actin expression from Vehicle, Days 0, 3, 7 after TNF‐α treatment (mean ± SEM; *p < 0.05, **p < 0.01, ***p < 0.001, ****p < 0.0001; *n* = 3). (C) Cryosection of Day 100 hSkMOs stained for PAX7, MYOD and DAPI (mean ± SEM; *p < 0.05, ***p < 0.001; *n* = 3). Scale bar = 50 μm. (D) Cryosection of Day 100 hSkMOs stained for PAX7, Ki67 and DAPI (mean ± SEM; *p < 0.05, ***p < 0.001; *n* = 3). Scale bar = 50 μm. (E) Cryosection of Day 100 hSkMOs stained for MYOG and DAPI (mean ± SEM; ***p < 0.001; *n* = 3). Scale bar = 100 μm. (F) Cryosection of Day 100 hSkMOs stained for MyHC, TUJ1 and αBTX (mean ± SEM; ****p < 0.0001; *n* = 20, ***p < 0.001, **p < 0.01; *n* = 3). Scale bar = 100 μm.

Sarcopenia is an age‐related condition characterized by the progressive loss of muscle mass, strength and function [1, 2, 5]. A key contributor to sarcopenia is chronic inflammation by pro‐inflammatory cytokines such as TNF‐α [22]. Previous studies have demonstrated that TNF‐α and NF‐*κ*B pathway activation promote age‐related changes and are associated with increased muscle degeneration, reduced protein synthesis and impaired muscle regeneration in sarcopenia due to muscle atrophy [23, 24, 25]. Treatments targeting TNF‐α in in vitro culture systems have been widely utilized in muscle atrophy and sarcopenia modelling studies [11, 22, 26]. Given the structural and functional maturity of the organoids and the presence of sustainable quiescent SCs within the basal lamina of muscle fibres, we expected our hSkMOs to serve as a modelling system for both muscle development and advanced skeletal muscle diseases such as sarcopenia. To optimize the TNF‐α treatment conditions for sarcopenia modelling, we first exposed hSkMOs to varying concentrations of TNF‐α (0, 5, 10, and 20 ng/mL) for 2 days and analysed the number of PAX7^+^ SCs by immunohistochemistry. The number of PAX7^+^ cells increased in a dose‐dependent manner, with the most pronounced activation observed at 20 ng/mL (Figure S4A). These results informed our decision to apply 20 ng/mL TNF‐α for the acute TNF‐α treatment in subsequent experiments. To investigate the acute pro‐inflammatory response and intrinsic regenerative capacity of day 100 hSkMOs possessing mature muscle tissue, we subjected them to TNF‐α treatment for 2 days and analysed the degree of muscle damage and regeneration at Days 0, 3, and 7 post‐treatments (Figure 6A). Western blot analysis confirmed that TNF‐α/NF‐*κ*B pathway associated factors such as NF‐*κ*B p65, I*κ*B‐α, and AKT were highly phosphorylated at Day 3 and drastically reduced at day 7 upon acute TNF‐α treatment (Figure 6B). Interestingly, we detected significantly increased expression of muscle differentiation‐associated genes, MYOG (Figure 6B), indicating activation of the NF‐*κ*B pathway by the acute TNF‐α treatment and induction of muscle regeneration.

Next, we conducted immunohistochemical analysis using antibodies against PAX7, MYOD, MYOG and Ki67 to assess the changes in SC status. The data revealed that approximately 13.62% of PAX7^+^/MYOD^+^ activated SCs drastically increased on day 0 and decreased over time (Figure 6C). We also observed 25.67% of PAX7^+^/Ki67^+^ proliferating SCs (Figure 6D). Additionally, the number of MYOG^+^ differentiated myocyte increased on Days 3 and 7 (Figure 6E). These data indicate that acute TNF‐α treatment in the hSkMOs culture system recapitulates the initiation of muscle wasting and activation of regenerative mechanisms in quiescent SCs. TNF‐α induces a transient activation of SCs, which then gradually revert to their baseline state, with increased proliferating and differentiating myogenic cells. We also assessed muscle fibre size using the MyHC marker to evaluate the impact of TNF‐α on muscle atrophy. The data showed a significant decrease in muscle fibre size on Days 0 and 3 post‐treatment (Figure 6F). However, the muscle fibres recovered to sizes comparable to those of the control, indicating that TNF‐α initially induces muscle atrophy but the muscle fibres regain their volume. Similarly, we analysed the number of αBTX^+^ NMJs and revealed a transient decrease in the number of NMJs following TNF‐α treatment, with gradual recovery over time to levels similar to those of the control (Figure 6F). Taken together, these results highlight the resilience and adaptive capacity of our hSkMOs, demonstrating their ability to recapitulate not only muscle degeneration but also the natural recovery process following pro‐inflammatory injury.

### 3.6 Testosterone Enhances SC Activation and Muscle Regeneration to Attenuate Muscle Wasting in hSkMOs Modelling Sarcopenia

**FIGURE 7:**
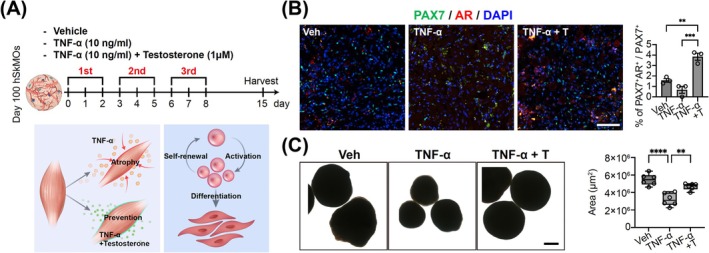
Sarcopenia modelling in hSkMOs and testosterone effects on attenuating muscle wasting. (A) A schematic diagram showing the overall strategy to chronic treatment of TNF‐α and testosterone on Day 100 hSkMOs. (B) Cryosection of Day 100 hSkMOs from each Vehicle, TNF‐α, and TNFα with Testosterone group stained for PAX7, androgen receptor (AR) and DAPI (mean ± SEM; **p < 0.01, ***p < 0.001; *n* = 3). Scale bar = 50 μm. (C) The representative images of hSkMOs from each Vehicle, TNFα, and TNFα with testosterone group (mean ± SEM; **p < 0.01, ****p < 0.0001; *n* = 6). Scale bar = 500 μm.

Several studies have explored the role of sex hormones, such as androgens and oestrogens, in the regulation and differentiation of quiescent SCs in skeletal muscle tissue homeostasis. Sex hormones have been shown to play an important role in inducing quiescent SCs for muscle regeneration [27]. In accordance with the acute pro‐inflammatory effect and immediate spontaneous regenerative capacity of mature hSkMOs (Figure 6), we next tested whether testosterone treatment of hSkMOs prevents muscle atrophy or ameliorates the progressive loss of muscle mass. To this end, we modelled the aging‐induced progressive deficit of muscle through chronic TNF‐α treatment (Figure 7A). This approach aimed to mimic the sustained inflammatory effect seen in sarcopenia and chronic muscle degeneration [28, 29]. Previous studies have reported that androgen receptors (ARs) are expressed in skeletal muscle cells, including myocytes and SCs [30, 31]. Consistent with these findings, ARs were predominantly expressed in PAX7^+^ SCs, and their expression was significantly increased by testosterone treatment (Figure 7B). Additionally, the proportion of PAX7^+^ SCs was significantly increased following co‐treatment with TNF‐α and testosterone, compared to that with TNF‐α treatment alone (Figure 7B). Based on the increased number of SCs observed after testosterone treatment, we speculated that loss of muscle mass could be prevented. We observed size reduction in TNF‐α‐treated hSkMOs, whereas the testosterone‐treated hSkMOs showed relatively similar sizes with the control hSkMOs (Figure 7C).

**FIGURE 8:**
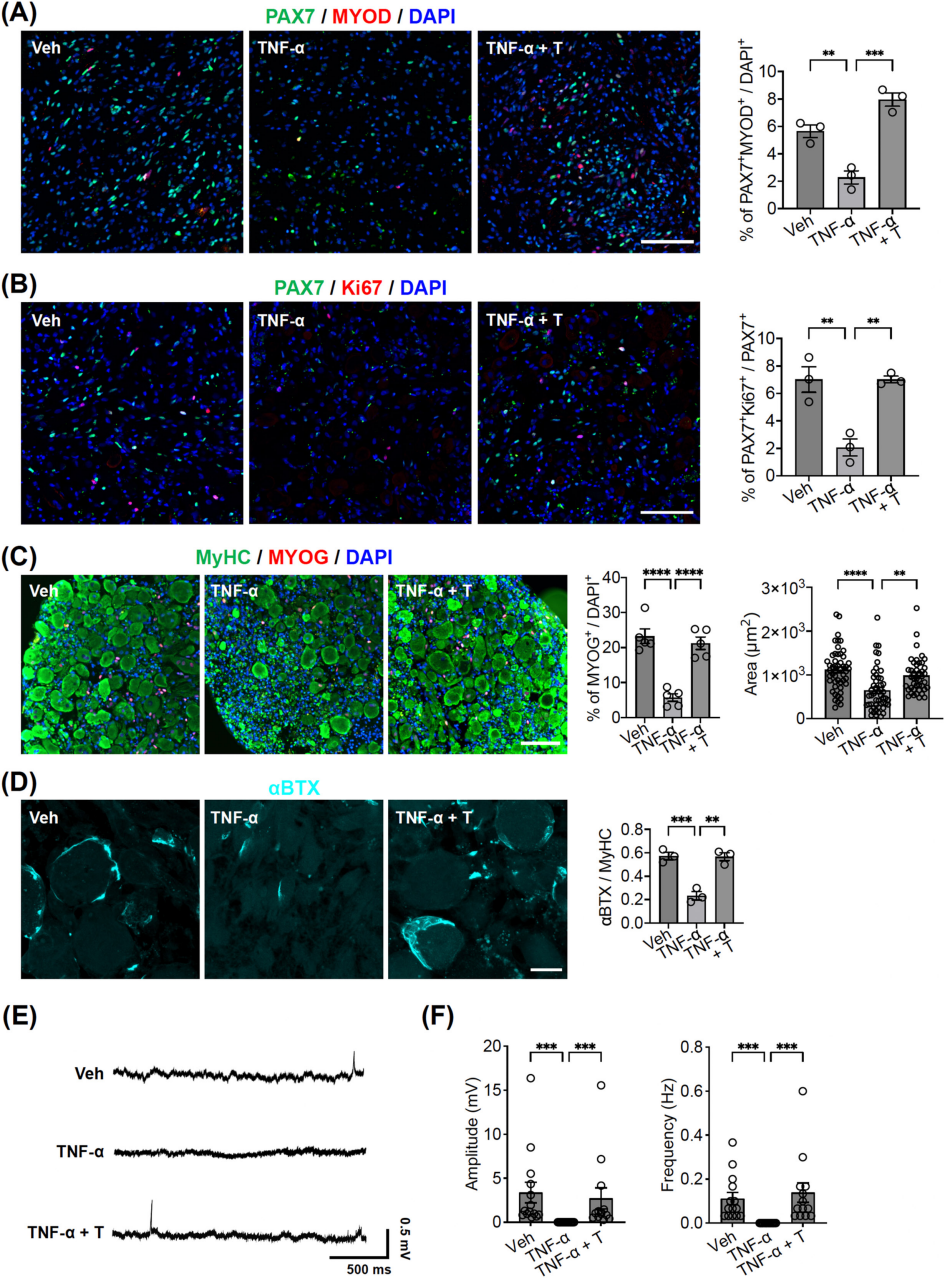
Regenerative capacity of hSkMOs upon testosterone treatment. (A) Cryosection of Day 100 hSkMOs from each Vehicle, TNF‐ɑ and TNFα with testosterone group stained for PAX7, MYOD and DAPI (mean ± SEM; **p < 0.01, ***p < 0.001; *n* = 3). Scale bar = 100 μm. (B) Cryosection of Day 100 hSkMOs from each Vehicle, TNF‐α and TNF‐α with testosterone group stained for PAX7, Ki67 and DAPI (mean ± SEM; **p < 0.01; *n* = 3). Scale bar = 100 μm. (C) Cryosection of Day 100 hSkMOs from each Vehicle, TNF‐α and TNF‐α with Testosterone group stained for MyHC, MYOG and DAPI (mean ± SEM; ****p < 0.0001; *n* = 5 and **p < 0.01; *n* = 50). Scale bar = 100 μm. (D) Cryosection of Day 100 hSkMOs from each Vehicle, TNF‐α and TNFα with testosterone group stained for αBTX (mean ± SEM; **p < 0.01, ***p < 0.001; *n* = 3). Scale bar = 20 μm. (E) Representative voltage traces of cells from the section of hSkMO subjected to various treatment conditions. (F) Co‐treatment with testosterone prevented the loss of postsynaptic potential observed in TNF‐α exposed cells (mean ± SEM; ***p < 0.001; Control: *n* = 14, TNF‐α: *n* = 14, TNF‐α + T: *n* = 13).

Next, we investigated whether the prevention of muscle atrophy was due to SC proliferation and differentiation to ensure muscle homeostasis and myogenesis. The hSkMOs were immunostained with antibodies against PAX7, MYOD, Ki67, MYOG and MyHC. The data revealed that the proportion of activated SCs (PAX7^+^/MYOD^+^, 7.97%; PAX7^+^/Ki67^+^, 7.03%) were significantly increased in the hSkMOs co‐treated with TNF‐α and testosterone compared to the hSkMOs treated with TNF‐α alone (Figure 8A,B). Consistently, we observed a significant increase in MYOG^+^ myocytes (21.23%) in the TNF‐α and testosterone‐co‐treated hSkMOs compared to that in MYOG^+^ myocytes (5.72%) in the TNF‐α treated hSkMOs without testosterone (Figure 8C). Furthermore, the size of MyHC^+^ muscle fibres increased due to the testosterone treatment (Figure 8C). Similarly, analysis of αBTX^+^ NMJs showed that chronic TNF‐α treatment alone resulted in a drastic reduction of NMJs. However, co‐treatment with testosterone effectively prevented this reduction, maintaining NMJ numbers comparable to that of control hSkMOs (Figure 8D). Furthermore, the presence of testosterone prevented the loss of membrane potential responses observed in TNF‐α‐treated samples (Figure 8E,F). These data indicate that testosterone may accelerate the activation and differentiation of SCs and preserve NMJ integrity, thereby preventing muscle atrophy caused by chronic inflammation.

## 4 Discussion

The development of a robust and physiologically relevant in vitro model of sarcopenia is crucial for understanding disease mechanisms and validating therapeutic interventions. In this study, we successfully established a 3D hSkMO model derived from hPSCs that recapitulates the key features of human skeletal muscle development, maturation, disease‐related degeneration and regeneration. By advancing our previous protocol for PMP differentiation, we achieved a stepwise transition from 2D paraxial mesodermal induction to 3D skeletal muscle maturation, which enabled the formation of well‐organized muscle fibres containing quiescent PAX7^+^ SCs beneath the basal lamina. This structure mimics the in vivo skeletal muscle microenvironment and offers a physiologically relevant platform for modelling muscle development and age‐related muscle loss, including sarcopenia.

Structural maturation was further supported by the formation of a well‐defined basal lamina that closely mimicked the in vivo skeletal muscle microenvironment. Morphologically, the muscle fibres exhibited mature, aligned, multinucleated muscle fibres similar to those observed in in vivo muscle tissues. The expression of key myogenic markers, such as MyHC and MYOG, along with the presence of quiescent PAX7^+^ SCs, underscores the developmental integrity of hSkMOs. Electrophysiological analysis confirmed the contractile capacity of the mature muscle fibres, highlighting the advanced functional maturation of hSkMOs. The presence of spontaneous contractions with robust calcium transients further reinforces the physiological relevance of this model. Moreover, whole‐cell patch clamp recordings revealed postsynaptic potentials, providing direct evidence of functional neuromuscular junction activity and synaptic communication within the hSkMOs. While these assessments confirm the presence of mature and electrically active neuromuscular components, they remain largely qualitative. To enhance the functional rigour of the platform, future efforts will focus on incorporating quantitative force measurement techniques such as muscular thin film assays or traction force microscopy to directly assess contractile strength and recovery dynamics in response to interventions like testosterone or injury. These quantitative metrics will provide an additional dimension for evaluating therapeutic efficacy and functional restoration [32]. Notably, these matured organoids harbour a diverse range of cell types, including myogenic cells, neural crest‐derived cells, glial cells and NMJ components. This cellular heterogeneity highlights the ability of hSkMOs to model the complex interactions within the neuromuscular system. Furthermore, the co‐existence of these distinct cell types within a single 3D system provides an opportunity to investigate cellular crosstalk in muscle development, degeneration and regeneration. The establishment of this cellular diversity is essential for studying NMJ formation and disease pathophysiology.

Our snRNA‐seq analysis provided an in‐depth transcriptional depiction of hSkMOs, unveiling their cellular composition and maturation. The data underscore the ability of hSkMOs to undergo progressive differentiation and structural organization, recapitulating key aspects of human myogenesis and neuromuscular development. A major finding of our study is the progressive maturation of myogenic progenitor/SCs toward myocytes and muscle fibres. This observation aligns with in vivo developmental paradigms in which the early myogenic population gives rise to mature, contractile muscle tissue as development proceeds [18]. The transcriptional downregulation of progenitor/SC markers and concomitant upregulation of myogenic fusion and fibre‐related genes reflect the intrinsic maturation of the muscle lineage within hSkMOs. Moreover, subclustering analysis revealed diverse SC status in quiescent, activated and differentiating SCs. In parallel, we observed neuronal and glial populations, including interneurons, motor neurons, astrocytes, and Schwann cells, demonstrating the multilineage potential of hSkMOs. Particularly, the emergence of diverse neuronal subtypes, including motor neurons, GABAergic and glutamatergic interneurons, further emphasizes the neural complexity captured within this organoid system. Together, these results confirm that hSkMOs not only replicate structural elements of skeletal muscle but also recapitulate the cellular heterogeneity and lineage dynamics characteristic of neuromuscular development. This comprehensive cellular architecture positions hSkMOs as a powerful platform for modelling muscle diseases, neural crest disorders, and neuromuscular interactions.

We established a senescence‐induced model using TNF‐α, a well‐known pro‐inflammatory cytokine linked to age‐related muscle atrophy. Acute TNF‐α exposure induced transient muscle atrophy followed by spontaneous recovery, highlighting the intrinsic regenerative capacity of SCs within the hSkMOs. This recovery was characterized by the reactivation of PAX7^+^ SCs, which underwent proliferation (PAX7^+^/Ki67^+^) and differentiation (PAX7^−^/MYOG^+^) to restore muscle mass and function. Additionally, chronic exposure to TNF‐α established a sustained sarcopenia‐like phenotype, characterized by persistent muscle atrophy, reduced muscle fibre volumes and functional decline. This is consistent with previous reports linking chronic inflammatory stress to long‐term muscle degeneration via the NF‐κB pathway [11, 33]. Our results demonstrated that chronic TNF‐α exposure leads to increased phosphorylation of NF‐*κ*B‐p65, promoting prolonged inflammatory signalling and impairing SC function. The persistent activation of NF‐*κ*B is known to drive catabolic pathways that degrade muscle proteins and inhibit muscle regeneration, leading to the progressive nature of sarcopenia. The sustained atrophy observed in the hSkMOs provides a valuable in vitro model for understanding the chronic effects of TNF‐α–mediated muscle atrophy and offers a platform for screening anti‐inflammatory therapeutic strategies.

Previous studies have shown that androgens, particularly testosterone, enhance muscle mass and strength by activating ARs on muscle cells, including SCs. Androgens stimulate SC proliferation and enhance muscle regeneration. This effect is mediated by AR expression, which increases the sensitivity of SCs to androgens [34, 35, 36]. In our hSkMO model, testosterone treatment significantly increased the expression of ARs, particularly in PAX7^+^ SCs, and preserved SC abundance and function. The presence of ARs suggests that testosterone directly influences SC proliferation and differentiation. This is supported by our findings that co‐treatment with TNF‐α and testosterone resulted in a marked increase in the proportion of activated SCs (PAX7^+^/MYOD^+^), proliferating SCs (PAX7^+^/Ki67^+^), and differentiating myogenic cells (MYOG^+^). These observations suggest that testosterone not only mitigates the negative effects of chronic inflammation on SCs but also actively promotes their regenerative potential. Moreover, muscle fibre size analysis revealed that testosterone treatment restored the volume of MyHC^+^ muscle fibres to levels comparable to that of untreated controls, even after chronic TNF‐α exposure. This finding is consistent with those of previous reports suggesting that testosterone stimulates muscle hypertrophy and promotes myogenic differentiation [35]. Collectively, these results suggest that testosterone is a potent anabolic agent capable of mitigating the deleterious effects of chronic inflammation on skeletal muscles by activating SCs and promoting muscle regeneration.

The key finding of our study is the regenerative capacity of SCs demonstrated in hSkMOs. Following acute TNF‐α exposure, SCs displayed the hallmark features of activation, proliferation, and differentiation, similar to the in vivo response to muscle injury. This response highlights the ability of hSkMOs to model muscle repair and regeneration. Importantly, we revealed that testosterone treatment enhances the regenerative response of SCs, promoting not only their activation and proliferation but also the formation of MYOG^+^ myogenic cells and MyHC^+^ muscle fibres. The ability of SCs to shift from a quiescent to an active state and subsequently revert to quiescence after repair is a crucial feature of muscle homeostasis. Our results demonstrate that hSkMOs harbour quiescent SCs beneath the basal lamina, similar to the in vivo skeletal muscle niche. Upon injury or inflammation induced by TNF‐α, these SCs activate, proliferate, and differentiate to restore muscle integrity. The subsequent return to a quiescent state reflects the intrinsic self‐renewal capacity of SCs, which is essential for long‐term muscle homeostasis. While our current results suggest a regenerative trajectory consistent with stemness, we acknowledge that direct evidence of long‐term self‐renewal such as lineage tracing or clonal expansion has not yet been obtained. Notably, our previous work demonstrated that PAX7^+^ cells in hSkMOs retained their identity and regenerative potential even after CTX‐induced injury, supporting their capacity to self‐renew in a damage‐recovery context [10]. To definitively establish stemness and quiescence re‐entry, it is necessary to utilize a genetic reporter system labelling SCs and long‐term clonal analysis in future studies.

The development of in vitro models to study sarcopenia is vital for advancing our understanding of the molecular mechanisms underlying age‐related muscle degeneration, particularly in response to cellular senescence. Senescence is a key contributor to sarcopenia progression, with senescent muscle cells losing their ability to regenerate and repair damaged tissue. Although numerous studies have linked chronic inflammation and oxidative stress to sarcopenia, the lack of efficient in vitro systems that recapitulate the progressive and multifactorial features of muscle aging has been a significant barrier to understanding these processes in detail [11]. The hSkMOs we developed offer a robust platform to model senescence‐induced muscle atrophy, as well as the regenerative potential of SCs, in response to inflammatory stimuli such as TNF‐α. By mimicking the effects of chronic inflammation on muscle tissue, our hSkMO model allows for the investigation of sarcopenia‐related molecular pathways, such as NF‐κB activation and their roles in muscle protein degradation and impaired regeneration [34]. Improving systems that induce senescence in muscle organoid models could enhance the accuracy and relevance of in vitro sarcopenia studies. While pro‐inflammatory cytokines such as TNF‐α have been successfully used to induce muscle wasting, these models often fail to fully replicate the long‐term inflammation characteristics of age‐related sarcopenia. To address this issue, it is crucial to refine senescence‐induced systems to closely mimic the chronic inflammatory environment in aging muscles. Approaches that involve extended exposure to TNF‐α or a combination of multiple inflammatory cytokines, in concert with oxidative stress, could better simulate the gradual onset of muscle atrophy observed in in vivo models. Additionally, the incorporation of cellular senescence markers, such as p16INK4a accumulation and β‐galactosidase activity, could further enhance the fidelity of these models, allowing for a more comprehensive study of the senescence‐associated secretory phenotype and its role in muscle degeneration.

In summary, we successfully developed a human organoid system to demonstrate the structural maturity of the skeletal muscle and its functional interaction with spinal‐derived motor neurons. Furthermore, we demonstrated that our system is useful for modelling aging‐induced sarcopenia. Our in vitro hSkMO model opens new avenues for understanding the underlying mechanisms of muscle degeneration and for developing novel therapeutic strategies.

## Conflicts of Interest

Ajou University School of Medicine has filed a provisional patent application that covers the generation of hSkMOs.

## Supporting information


**Table S1:** List of antibodies.
**Table S2:** List of reagents.
**Table S3:** Primer sequences.
**Figure S1:** (A) The gene expression of pPSM (*T/BRA, MSGN1, TBX6*), aPSM (*PAX3*), somite (*MEOX2*) markers at different time points (Days 0, 3, 6 and 9) of hSkMOs were identified by quantitative real‐time reverse transcription‐polymerase chain reation (qRT‐PCR) analysis. The qRT‐PCR data were normalized to *GAPDH* expression (mean ± SEM; *n* = 3). **p < 0.01, ***p < 0.001, ****p < 0.0001 (B) Cryosection of Day 15 hSkMOs stained for TFAP2A, SOX2 and DAPI. Scale bar = 50 μm. (C) Cryosection of Day 15 hSkMOs stained for PAX7, SOX2 and DAPI. Scale bar = 50 μm.
**Figure S2:** (A) Representative brightfield images of hSkMOs derived from H9 hESCs and CHA‐SCNT‐PSC‐18 hPSCs at Days 10, 30, 50 and 100. Scale bar = 500 μm. The growth diameter demonstrates the average size (mean ± SEM; *n* = 3). (B) Cryosection of Day 30 hSkMOs stained for PAX7 and MYOD. Scale bar = 100 μm. Quantification of PAX7^+^/MYOD^−^ quiescent SC and PAX7^+^/MYOD^+^ activated SCs in hSkMO‐derived from both cell lines (mean ± SEM; *n* = 3). (C) Cryosection of Day 100 hSkMOs stained for MyHC, TUJ1 and DAPI. Scale bar = 500 μm and Quantification of the proportion of TUJ1^+^ neural and MyHC^+^ muscle region in H9‐ and CHA‐SCNT‐PSC‐18‐derived hSkMOs (mean ± SEM; *n* = 3). (D) Cryosection of Day 50 hSkMOs stained for MyHC, PDGFRɑ, and DAPI. Scale bar = 500 μm.
**Figure S3:** (A) Feature plots showing gene expression of selected markers. (B) Feature plots showing gene expression of myogenic progenitors/SCs subclusters. (C) Feature plots showing gene expression of neural subclusters.
**Figure S4:** (A) Cryosection of Day 100 hSkMOs stained for PAX7 and DAPI (mean ± SEM; *p < 0.05, **p < 0.01, ****p < 0.0001; *n* = 3). Scale bar = 100 μm.
