# Supplementary material for: Human Pluripotent Stem Cell‐Derived Skeletal Muscle Organoid Model of Aging‐Induced Sarcopenia
Source: J Cachexia Sarcopenia Muscle. 2025 Aug 14;16(4):e70045. doi: 10.1002/jcsm.70045 (PMC12351639; doi:10.1002/jcsm.70045)
Supplement: Supplementary file 1 — Table S1: List of antibodies. Table S2: List of reagents. Table S3: Primer sequences. Figure S1: (A) The gene expression of pPSM (T/BRA, MSGN1, TBX6), aPSM (PAX3), somite (MEOX2) markers at different time points (Days 0, 3, 6 and 9) of hSkMOs were identified by quantitative real‐time reverse transcription‐polymerase chain reation (qRT‐PCR) analysis. The qRT‐PCR data were normalized to GAPDH expression (mean ± SEM; n = 3). **p < 0.01, ***p < 0.001, ****p < 0.0001 (B) Cryosection of Day 15 hSkMOs stained for TFAP2A, SOX2 and DAPI. Scale bar = 50 μm. (C) Cryosection of Day 15 hSkMOs stained for PAX7, SOX2 and DAPI. Scale bar = 50 μm. Figure S2: (A) Representative brightfield images of hSkMOs derived from H9 hESCs and CHA‐SCNT‐PSC‐18 hPSCs at Days 10, 30, 50 and 100. Scale bar = 500 μm. The growth diameter demonstrates the average size (mean ± SEM; n = 3). (B) Cryosection of Day 30 hSkMOs stained for PAX7 and MYOD. Scale bar = 100 μm. Quantification of PAX7+/MYOD− quiescent SC and PAX7+/MYOD+ activated SCs in hSkMO‐derived from both cell lines (mean ± SEM; n = 3). (C) Cryosection of Day 100 hSkMOs stained for MyHC, TUJ1 and DAPI. Scale bar = 500 μm and Quantification of the proportion of TUJ1+ neural and MyHC+ muscle region in H9‐ and CHA‐SCNT‐PSC‐18‐derived hSkMOs (mean ± SEM; n = 3). (D) Cryosection of Day 50 hSkMOs stained for MyHC, PDGFRɑ, and DAPI. Scale bar = 500 μm. Figure S3: (A) Feature plots showing gene expression of selected markers. (B) Feature plots showing gene expression of myogenic progenitors/SCs subclusters. (C) Feature plots showing gene expression of neural subclusters. Figure S4: (A) Cryosection of Day 100 hSkMOs stained for PAX7 and DAPI (mean ± SEM; *p < 0.05, **p < 0.01, ****p < 0.0001; n = 3). Scale bar = 100 μm. [file JCSM-16-e70045-s001.docx]

**Supplementary Information**

**Human pluripotent stem cell-derived skeletal muscle organoid model of aging-induced sarcopenia**

Seongjun Park, Min-Kyoung Shin, Dong Seok Jeong, Xin Yi Yeo, Yeo Jin Kim, Junaid Muhammad, Minju Kim, Seongeun Gu, Jeoung Eun Lee, Yunjin Park, Su Bin Lim, Ji Young Mun, Sangyong Jung, Dong Ryul Lee, Junghyun Jo

1. **Supplementary Methods**
2. **Supplementary Tables**
3. **Supplementary Figures**

**Supplementary Methods**

***Culture of hPSCs***

The H9 (WA09; WiCell Research Institute), H1 (WA01; WiCell Research Institute), and CHA-SCNT-PSC-18 (Korea Stem Cell Registry code: hES12019001), which was previously established by our group [1]. The hPSCs were cultured on Matrigel-coated plates (Corning) in mTeSR medium (Stem Cell Technologies) at 37°C in a humidified 5% CO_2_ incubator. Cell lines were validated for normal karyotype and were mycoplasma free. The medium for hPSCs was refreshed daily and cells were passaged every 5-6 days.

***Immunofluorescence analyses using cryosections***

hSkMOs were carefully washed 3 times in phosphate-buffered saline (PBS). Subsequently, the hSkMOs were fixed with 4% paraformaldehyde (PFA) overnight. Following fixation, the hSkMOs were washed with PBS and then immersed in a PBS containing 30% sucrose. The hSkMOs were embedded in O.C.T. compound for snap-frozen using isopentane pre-chilled with liquid nitrogen and stored in -80°C. hSkMOs were sectioned into 10 μm thick sections using a cryostat (Leica CM1950). Following this, the cryosectioned slices were washed with PBS. The slices were blocked with 5% Normal Donkey Serum (NDS), 2% Bovine Serum Albumin (BSA) (Sigma Aldrich) and 0.2% Triton X-100 (Sigma Aldrich) in PBS for 1 hour at room temperature and incubated with primary antibodies overnight at 4°C. Slides were then washed 3 times with 0.1% Triton X-100 (Sigma Aldrich) in PBS and incubated with secondary antibodies and DAPI for each 1 hour and 5 minutes at room temperature. `Slides were mounted with mounting solution (biomeda).

***RNA extraction, reverse transcription, and real-time quantitative RT-PCR***

To extract total RNA from hSkMOs, the RNAiso Plus kit (Takara Bio Inc., Shiga, Japan) was used, which contains phenol. The isolated RNA (1 μg) was then reverse transcribed into cDNA using TOPscriptTM RT DryMIX (dT18 plus) from Enzynomics Inc. (Daejeon, Korea) and the T100TM Thermal Cycler from Bio-Rad (Hercules, CA), following the manufacturer's instructions. For quantitative reverse transcription polymerase chain reaction (qRT-PCR) analysis, amplification reactions were performed using the SYBR Green TOPrealTM qPCR 2X PreMIX (Enzynomics), following the manufacturer's protocol. The reactions were carried out using the CFX96TM Real-Time System and C1000TM Touch Thermal Cycler (Bio-Rad).

**Library preparation for single nucleus RNA sequencing**

Single-nucleus RNA-seq libraries were prepared using the Chromium GEM-X Single Cell 3' Kit v4 (PN-1000691) (10x Genomics, CA) and Chromium X instrument, following the manufacturer's protocol (CG000731, Rev B). Briefly, nuclei suspensions were mixed with reverse transcription master mix and loaded into a Chromium GEM-X Single Cell 3' Chip Kit v4 (PN-1000690) (10x Genomics, CA) along with Single Cell 3′ Gel Beads and Partitioning Oil to generate Gel Bead-in-Emulsions (GEMs). Within each GEM, polyadenylated mRNA transcripts were uniquely barcoded and reverse transcribed. Following reverse transcription, GEMs were broken, and barcoded full-length cDNA was purified and amplified via PCR. The amplified cDNA was then subjected to enzymatic fragmentation, end-repair, A-tailing, adaptor ligation, and sample index PCR to construct 3’ gene expression libraries. The quality of amplified libraries was confirmed by electrophoresis on Agilent Bioanalyzer High Sensitivity DNA Kit (part # 5067-4626) (Agilent, CA). Libraries were quantified using the KAPA Library Quantification Kit (KK4824) (Kapa Biosystems, MA) according to the manufacturer’s library quantification protocol. Following cluster amplification of denatured templates, sequencing was progressed as paired-end (2×150bp) using Illumina Novaseq X plus (Illumina, CA).

**Single nucleus transcriptome analysis**

Raw gene expression matrix files from day 50 hSkMO samples were loaded using Read10X function in Seurat (v. 5.3.0). Seurat objects were created using the CreateSeuratObject function by retaining the cells with at least 200 detected genes per cell and 3 cells per gene. To ensure the quality of data analyses, cells with feature count over 5,500 and less than 300 were filtered out. A threshold of <10% mitochondrial content was chosen based on the observed distribution of mitochondrial percentages. Filtered Seurat objects from both timepoint were merged and standard normalization and variance stabilization were applied followed by the identification of highly variable genes. We then performed the principal component analysis (PCA) followed by UMAP visualization to assess the batch differences. To correct the timepoint specific batch effects, we applied the IntegrateLayers using canonical correlation analysis (CCA). The integrated data was then re-clustered using 20 principal components along with UMAP embeddings to visualize the clusters in a shared space.

***Western blot***

hSkMOs were harvested and homogenized in PRO-PREPTM Protein Extraction Solution (Intron Biotech) supplemented with phosphatase and protease inhibitor cocktails (Roche) to preserve phosphorylated proteins. Following centrifugation at 13,000 rpm for 5 minutes at 4°C, the supernatant was collected. Protein samples (20 μg) were separated on 8–12% SDS-PAGE gels and transferred to PVDF membranes (Bio-Rad). Membranes were blocked with 5% BSA for 1 hour at room temperature and incubated overnight at 4°C with primary antibodies against phospho-NF-κB p65 (Ser536) (Cell Signaling, A19653, 1:1000), phospho-IκBα (Ser32) (Cell Signaling, #2859, 1:300), phospho-Akt (Ser473) (Cell Signaling, #9271, 1:1000), and MYOG (Abcam, ab124800, 1:1000). After washing, membranes were incubated with HRP-conjugated anti-mouse or anti-rabbit secondary antibodies (Cell Signaling, #7074, #7076, 1:5000) and visualized using SuperSignal West Femto Maximum Sensitivity Substrate (Thermo Fisher). Membranes were stripped using Easter-Blot™ Western Blot Stripping Buffer (Biomax) and reprobed with β-actin (Cell Signaling, sc-47778, 1:20000). Band intensities were quantified using Image J software.

***Contraction analysis***

Video segments of equal length were recorded using a brightfield microscope (DMI1, Leica) in 3 different regions of the same organoid using 20X zoom. To quantify the muscle contraction, each movie was analyzed individually using MuscleMotion (an open-source ImageJ Macro). A reference frame was automatically detected by MuscleMotion to measure the contraction as a variation in pixel over time. The results were then plotted in GraphPad Prism 10.

***Whole-cell patch clamp recordings***

For functional analyses of the organoids, we sectioned each organoid to 300 μm thickness slices using a vibratome (Leica CT1200S). Each slice was placed and maintained on Matrigel coated glass coverslips in a 5% CO2 incubator at 37°C, with medium including MMM media, till use for functional analysis. Glass coverslips containing the cultured organoid slice are transferred to the recording chamber constantly perfused with recording solution containing the following (in mM): 140 NaCl, 10 D-glucose, 10 HEPES, 2.4 KCl, 2 CaCl_2_, and 1 MgCl_2_ (300-310 mOsm, pH 7.3-7.4 with NaOH). Pipettes used for recording were pulled from borosilicate glass capillary tubes with filament (length 100 mm, outer diameter 1.5 mm, inner diameter 0.84 mm, WPI) using a P-1000 Flaming/Brown Micropipette Puller (Sutter Instruments).Patch pipettes (5-7 MΩ) were filled with internal solution containing (in mM) 135 K-Gluconate, 4 NaCl, 10 HEPES, 2 MgCl_2_, 2 NaATP, 0.3 NaGTP, 0.06 EGTA, and 0.01 CaCl_2_. Cells are visualised using an optiMOS scientific CMOS camera (Iris 9, Teledyne Photometrics) and monitor. Recordings were obtained with Clampfit 11 and the signals were amplified by Multiclamp-700A amplifier (Axon Instruments), digitized by Digidata-1550B (Axon Instruments).

***Calcium imaging***

For calcium imaging experiments day 100 hSkMOs were incubated at 37°C for 1 hour in MMM medium (without phenol red) containing 5μM of the cell-permeable calcium indicator Fluo-4 AM and 0.02% Pluronic F-127 (diluted in 20% DMSO). Staining was performed in the dark, followed by two washes in MMM medium (without phenol red). The organoid was then left to recover for 15 min prior to recording. Images were captured with a 100 ms exposure time (10 Hz). A standard-deviation image was generated to detect pixels displaying the most variable intensity during the recording. Individual or small cell clusters were automatically identified and the *Δ*F/F variation over time was plotted for the defined regions of interest.

***Scanning electron microscopy (SEM)***

The hSkMOs were fixed in 2% paraformaldehyde and 2.5% glutaraldehyde in 0.15 M cacodylate buffer (pH 7.4). After one day, they were embedded in 3% agarose and sectioned at 150 μm using a vibratome (Leica, VT1000S). After washing, they were post-fixed with 2% osmium tetroxide (OsO_4_)/1.5% potassium ferrocyanide (EMS/ Sigma) for 1 h on ice and washed several times. They were placed in 1% thiocarbohydrazide (TCH) (Sigma) solution for 20 min and then transferred to 2% aqueous OsO4 for 30 min. They were incubated in 1% uranyl acetate (EMS) overnight. After washing, they were incubated in lead aspartate solution for 30 min. They were dehydrated using a graded series of ethanol (20%, 50%, 70%, 90%, and 100%) on ice and infiltrated with 100% ethanol and 100% acetone, a mixture of resin and acetone, and 100% acetone. The resin was prepared with the Epon 812 kit (EMS) following the manufacturer’s instructions. Samples were placed in embedding tubes with fresh Epon mixture at 60°C for 2 days. For SEM imaging, the embedded samples were trimmed, and 70 nm sections were cut from the block using an ultramicrotome with a diamond knife. The sections were mounted onto indium-tin-oxide (ITO)-coated coverslips. Images were imaged using ZEISS Atlas5 software for the Gemini 300 SEM (ZEISS) at an accelerating voltage of 5 kV.

***TNF-α and testosterone treatment of hSkMOs***

For acute inflammatory stimulation, hSkMOs at day 100 were treated with recombinant human TNF-α (PeproTech) at 20 ng/mL for two consecutive days. Following acute TNF-α treatment, hSkMOs were harvested at day 0, day 3, and day 7. For chronic inflammatory modeling, hSkMOs were exposed to 10 ng/mL TNF-α through three repeated cycles (Days 0 – 2, 3 – 5, and 6 – 8), with each cycle consisting of two days of treatment followed by one day without TNF-α. hSkMOs were harvested at day 7 after completion of the final treatment cycle. Throughout the treatment periods, hSkMOs were maintained in MMM medium on an orbital shaker to ensure even distribution of TNF-α and the culture medium was refreshed every three days. To evaluate the protective effects against chronic TNF-α treatment, 1 μM testosterone (Sigma-Aldrich) was co-treated during chronic TNF-α treatment cycles.

***Analysis of muscle fiber diameter and NMJ density***

For muscle fiber diameter analysis, MyHC-stained cross-sectional images were used. The minimal Feret's diameter was measured at three different positions within each muscle fiber, and the average value was calculated to represent the fiber diameter [2]. For NMJ density analysis, αBTX-stained images were analyzed by determining whether each individual MyHC⁺ muscle fiber was associated with an αBTX⁺ NMJ, and the percentage of NMJ-positive fibers was calculated relative to the total number of analyzed muscle fibers in the same field of view [3]. All image analyses were performed using Image J, and all images were acquired under the same exposure and gain settings. Analysis parameters were kept consistent across all experimental groups.

**Supplementary Tables**

**Supplementary Table 1. List of antibodies**

| **Antibodies** | **Company** | **Cat.#** |
| --- | --- | --- |
| Goat anti-ChAT | Millipore | Cat.No.: AB144P; RRID: AB_2079751 |
| Goat anti-T/BRA | R&D | Cat.No.: AF2085; RRID: AB_2200235 |
| Goat anti-TBX6 | R&D | Cat.No.: AF4744; RRID: AB_2200834 |
| Rabbit anti-SOX2 | Millipore | Cat.No.: AB5603; RRID: AB_2286686 |
| Mouse anti-TFAP2A | DSHB | Cat.No.: 3B5-c  RRID: AB_528084 |
| Mouse anti-PAX7 | DSHB | Cat.No.: PAX7-c  RRID: AB_528428 |
| Goat anti-SOX10 | R&D | Cat.No.: AF2864  RRID: AB_442208 |
| Rabbit anti-MYOG | Abcam | Cat.No.: AB124800 RRID: AB_10971849 |
| Rabbit anti-MYOD | Abcam | Cat.No.: AB133627 RRID: AB_2890928 |
| Rabbit anti-TUJ1 | GeneTex | Cat.No.: GTX130245 RRID: AB_2886220 |
| Chicken anti-MAP2 | Abcam | Cat.No.: AB5392  RRID: AB_2138153 |
| Rabbit anti-Androgen receptor | Invitrogen | Cat.No.: PA5-85072  RRID: AB_2792221 |
| Mouse anti-PAX3 | DSHB | Cat.No.: PAX3  RRID: AB_528426 |
| Rabbit anti-Ki67 | Abcam | Cat.No.: ab15580  RRID: AB_443209 |
| Mouse anti-Fast MyHC | Sigma-Aldrich | Cat.No.: M1570  RRID: AB_2147168 |
| Rabbit anti-Laminin | Abcam | Cat.No.: AB11575  RRID: AB_298179 |
| Rabbit anti-GFAP | Sigma Aldrich | Cat.No.: SAB5600060 |
| Rabbit anti-S100β | Abcam | Cat.No.: AB52642  RRID: AB_882426 |
| Alexa 647 Conjugate α-bungarotoxin | Thermo Fisher | Cat.No.: B35450 |
| Phospho-NF-kB p65 (Ser536) | Cell Signaling Technology | Cat.No.: A19653  RRID: AB_331284 |
| Phospho-IκBα (Ser32) | Cell Signaling Technology | Cat.No.: 2859S  RRID: AB_561111 |
| Phospho-Akt | Cell Signaling Technology | Cat.No.: 9271S  RRID: AB_329827 |
| β-actin | Santa Cruz Biotechnology | Cat.No.: sc-47778  RRID: AB_626632 |
| Anti-mouse IgG, HRP-linked Antibody | Cell Signaling Technology | Cat.No.: 7076S  RRID: AB_330924 |
| Anti-rabbit IgG, HRP-linked Antibody | Cell Signaling Technology | Cat.No.: 7074S  RRID: AB_2099233 |
| Donkey anti-mouse-Alexa 488 | Invitrogen | Cat.No.:A-21202; RRID: AB_141607 |
| Donkey anti-rabbit-Alexa 568 | Invitrogen | Cat.No.:A10042;  RRID: AB_2534017 |
| Donkey anti-goat-Alexa 488 | Invitrogen | Cat.No.: A-11055; RRID: AB_2534102 |
| Donkey anti-goat-Alexa 647 | Invitrogen | Cat.No.: A-21447; RRID: AB_2535864 |
| Goat anti-chicken-Alexa 647 | Invitrogen | Cat.No.: A-21449; RRID: AB_2535866 |

**Supplementary Table 2. List of reagents**

| **Chemicals, peptides, and recombinant proteins** | **Company** | | **Cat.#** |
| --- | --- | --- | --- |
| mTESR1 plus | StemCellTechnologies | 85875 | |
| DMEMF-12 | LifeTechnologies | 11320033 | |
| MEMNon-EssentialAminoAcids | LifeTechnologies | 11140050 | |
| Penicillin-Streptomycin | LifeTechnologies | 15140122 | |
| GlutaMAX | LifeTechnologies | 35050061 | |
| β-Mercaptoethanol | LifeTechnologies | 31350010 | |
| N2 | LifeTechnologies | 17502001 | |
| B27 Supplement (50X), minus vitamin A | LifeTechnologies | 12587010 | |
| Matrigel | Corning | 354277 | |
| TrypLE Express | LifeTechnologies | 12604013 | |
| Y-27632 | Tocris Bioscience | 1254 | |
| CHIR99021 | StemCellTechnologies | 252917-06-9 | |
| LDN193189 | Stemgent | 04-0074 | |
| bFGF | Peprotech | 100-18B | |
| HGF | Peprotech | 100-39H | |
| IGF-1 | Sigma-Aldrich | I3769 | |
| SB-431542 | Sigma-Aldrich | 301836-41-9 | |
| Insulin-Transferrin-Selenium | LifeTechnologies | 41400-045 | |
| KnockOut Serum Replacement (SR) | LifeTechnologies | 10828028 | |
| TNF-α | Peprotech | 300-01A | |
| Testosterone | Millipore | 58-22-0 | |
| Sucrose | Sigma-Aldrich | 57-50-1 | |
| DAPI | Sigma-Aldrich | D9542 | |
| BovineSerumAlbumin | Bovogen | BSAS 0.1 | |
| Pluronic F-127 | Sigma Aldrich | 9003-11-6 | |
| Fluo-4, AM, cell permeant | Invitrogen | F14201 | |

**Supplementary Table 3. Primer sequences**

| **Name** | **Pimer** | |
| --- | --- | --- |
| T/BRA F | 5’-TTCATAGCGGTGACTGCTTATCA-3’ |  |
| T/BRA R | 5’-CACCCCCATTGGGAGTACC-3’ |  |
| MSGN1 F | 5’-CTGCACACCCTCCGGAATT-3’ |  |
| MSGN1 R | 5’-CTCTGCCGCGGTTAAGGAG-3’ |  |
| TBX6 F | 5’-CATCCACGAGAATTGTACCCG-3’ |  |
| TBX6 R | 5’-AGCAATCCAGTTTAGGGGTGT-3’ |  |
| PAX3 F | 5’-AGCTCGGCGGTGTTTTTATCA-3’ |  |
| MEOX2 F | 5’-CTCTGCAAACCAACTGGCAC-3’ |  |
| MEOX2 R | 5’-AAGAGTTGGAGCACAGGACG-3’ |  |
| GAPDH F | 5’-CAAGATCATCAGCAATGCCTCCTG-3’ |  |
| GAPDH R | 5’-GCCTGCTTCACCACCTTCTTGA-3’ |  |

**Supplementary Figures**

**Supplementary Figure 1**


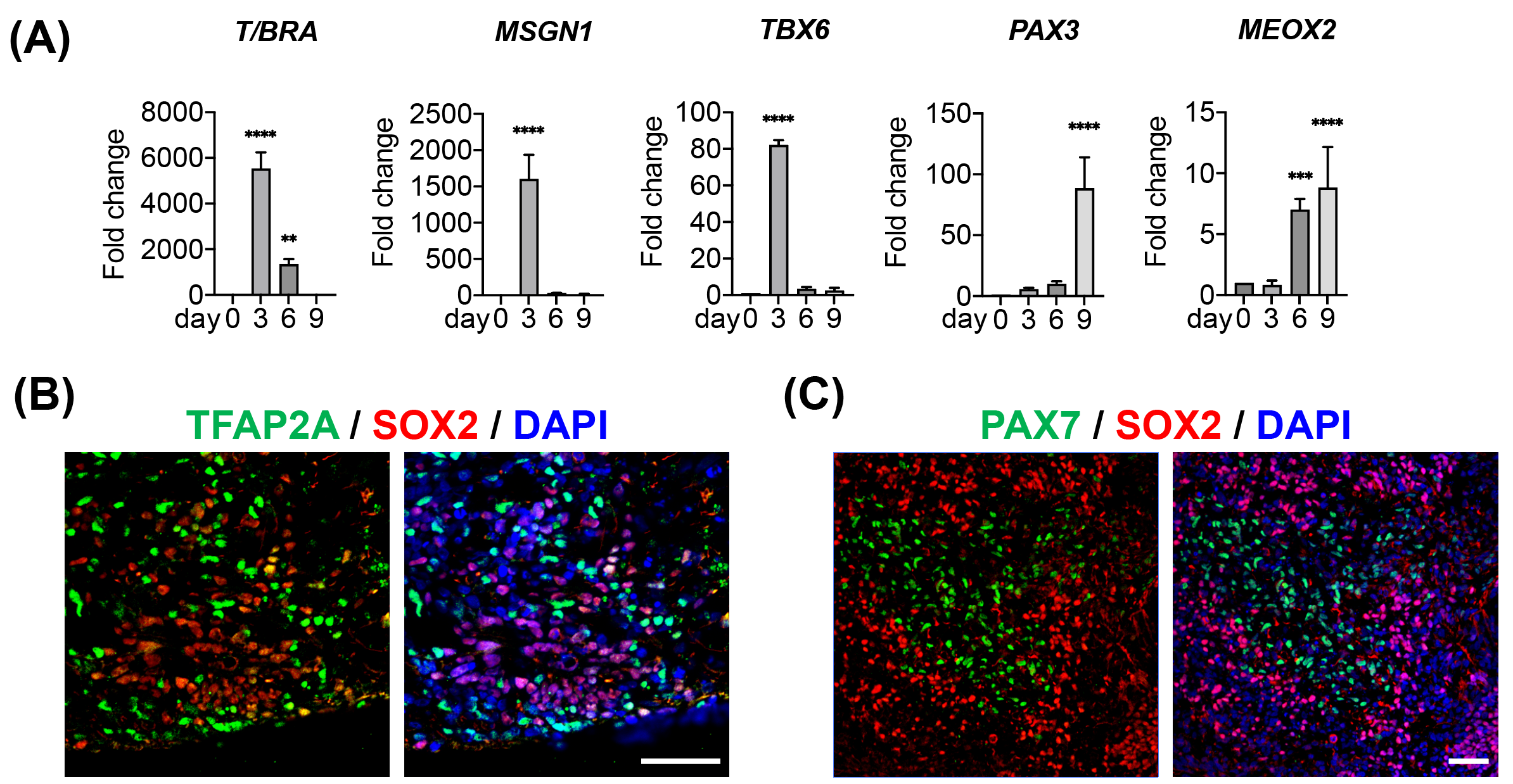


**Figure S1.** (A) The gene expression of pPSM (*T/BRA, MSGN1, TBX6*), aPSM (*PAX3*), somite (*MEOX2*) markers at different time points (days 0,3,6, and 9) of hSkMOs were identified by quantitative real-time reverse transcription-polymerase chain reation (qRT-PCR) analysis. The qRT-PCR data were normalized to *GAPDH* expression. (mean ± SEM; n = 3). ** *p* < 0.01, *** *p* < 0.001, **** *p* < 0.0001 (B) Cryosection of day 15 hSkMOs stained for TFAP2A, SOX2, and DAPI. Scale bar = 50 µm. (C) Cryosection of day 15 hSkMOs stained for PAX7, SOX2, and DAPI. Scale bar = 50 µm.

**Supplementary Figure 2**

**
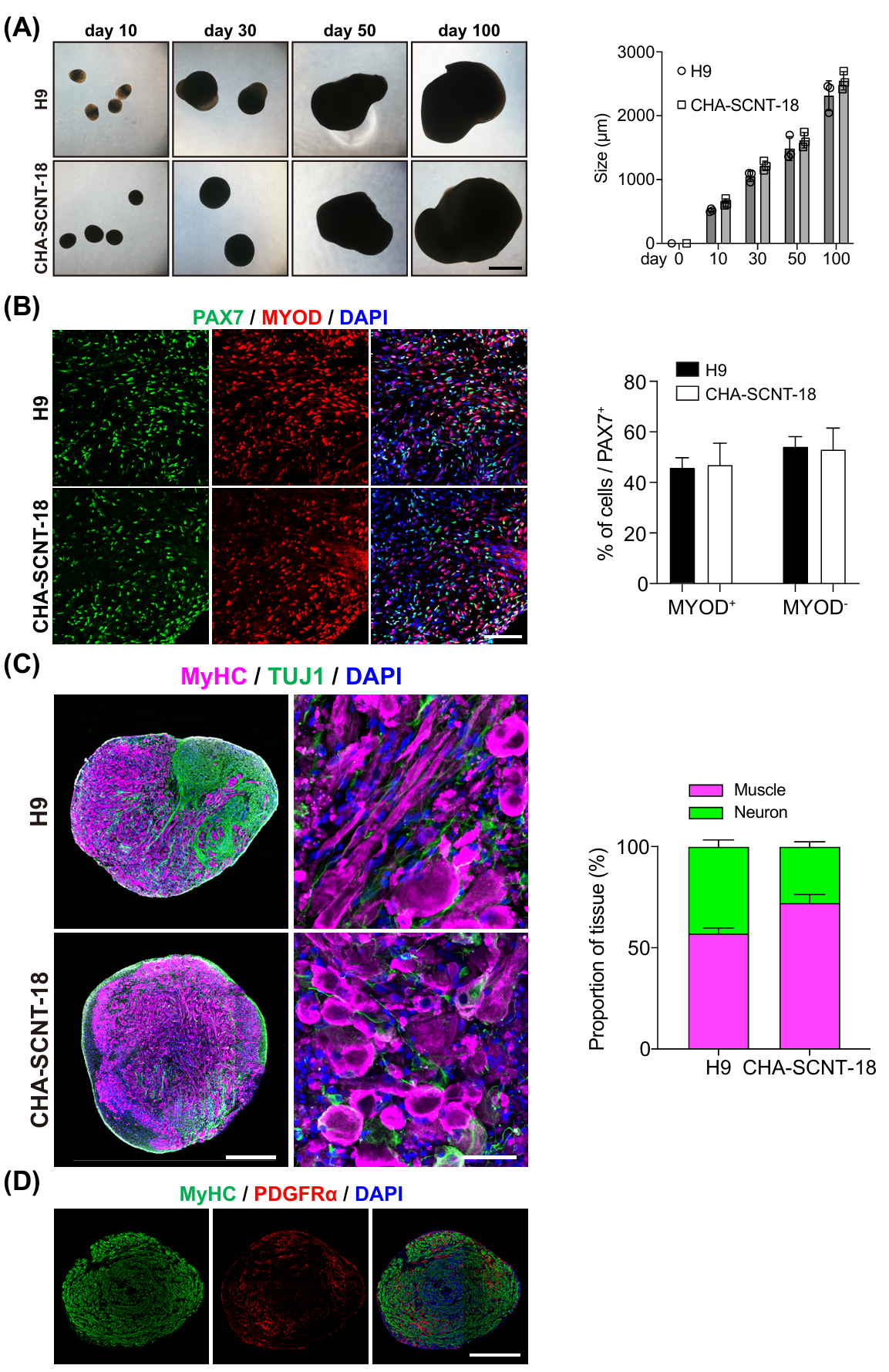
**

**Figure S2.** (A) Representative brightfield images of hSkMOs derived from H9 hESCs and CHA-SCNT-PSC-18 hPSCs at days 10, 30, 50, and 100. Scale bar = 500 µm. The growth diameter demonstrates the average size (mean ± SEM; n = 3). (B) Cryosection of day 30 hSkMOs stained for PAX7 and MYOD. Scale bar = 100 µm. Quantification of PAX7⁺/MYOD⁻ quiescent SC and PAX7⁺/MYOD⁺ activated SCs in hSkMO-derived from both cell lines (mean ± SEM; n = 3). (C) Cryosection of day 100 hSkMOs stained for MyHC, TUJ1, and DAPI. Scale bar = 500 µm and Quantification of the proportion of TUJ1^+^ neural and MyHC^+^ muscle region in H9- and CHA-SCNT-PSC-18-derived hSkMOs (mean ± SEM; n = 3). (D) Cryosection of day 50 hSkMOs stained for MyHC, PDGFRɑ, and DAPI. Scale bar = 500 µm.

**Supplementary Figure 3**

**
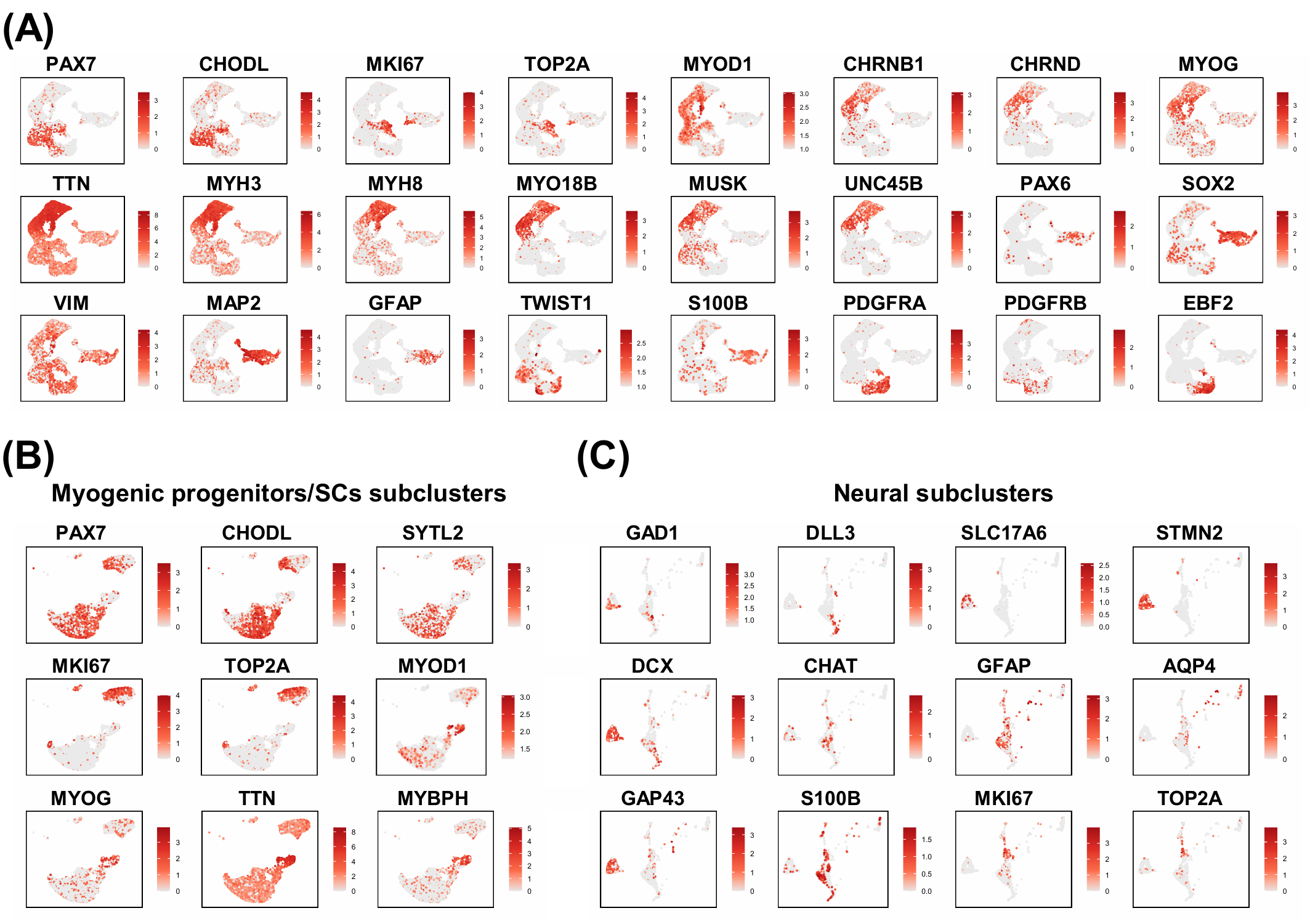
**

**Figure S3.** (A) Feature plots showing gene expression of selected markers. (B) Feature plots showing gene expression of myogenic progenitors / SCs subclusters. (C) Feature plots showing gene expression of neural subclusters.

**Supplementary Figure 4**


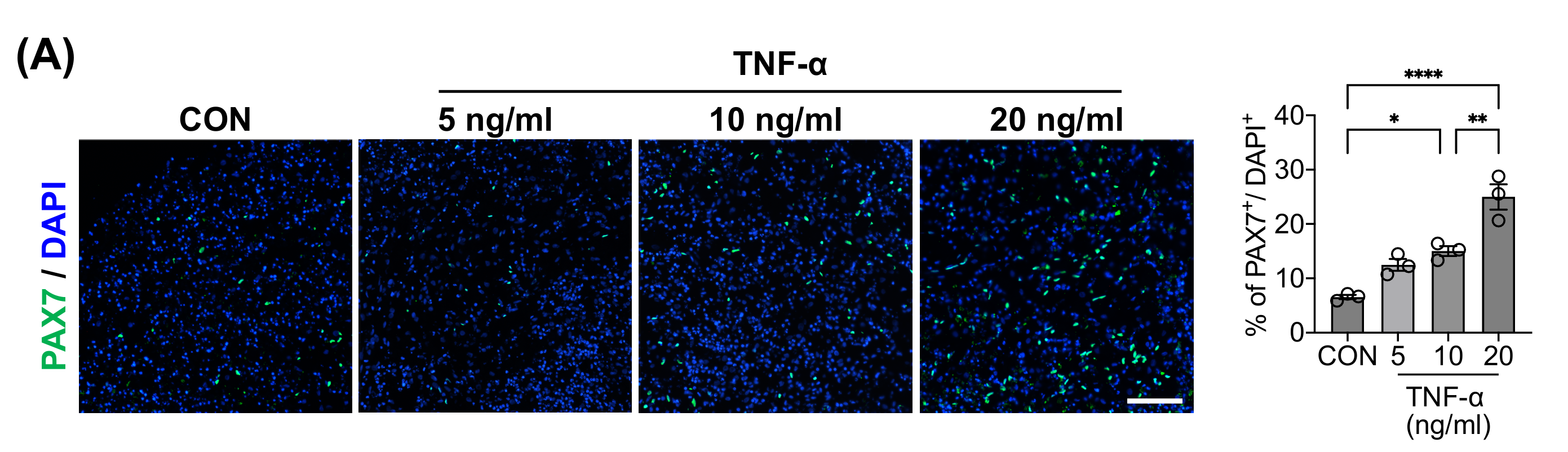


**Figure S4.** (A) Cryosection of day 100 hSkMOs stained for PAX7 and DAPI (mean ± SEM; **p* < 0.05, ***p* < 0.01, *****p* < 0.0001; n = 3). Scale bar = 100 µm.

**REFERENCES**

1. Lee JE, Lee JY, Park CH, Eum JH, Jung SK, Han AR, et al. Cryopreserved Human Oocytes and Cord Blood Cells Can Produce Somatic Cell Nuclear Transfer-Derived Pluripotent Stem Cells with a Homozygous HLA Type. Stem Cell Reports. 2020;15:171-84. doi:10.1016/j.stemcr.2020.05.005

2. Briguet A, Courdier-Fruh I, Foster M, Meier T, Magyar JP. Histological parameters for the quantitative assessment of muscular dystrophy in the mdx-mouse. Neuromuscul Disord. 2004;14:675-82. doi:10.1016/j.nmd.2004.06.008

3. Faustino Martins JM, Fischer C, Urzi A, Vidal R, Kunz S, Ruffault PL, et al. Self-Organizing 3D Human Trunk Neuromuscular Organoids. Cell Stem Cell. 2020;26:172-86 e6. doi:10.1016/j.stem.2019.12.007
